# Integrative analysis based on the cell cycle-related genes identifies TPX2 as a novel prognostic biomarker associated with tumor immunity in breast cancer

**DOI:** 10.18632/aging.205752

**Published:** 2024-04-19

**Authors:** Xinli Liu, Wenyi Wang, Bing Chen, Shengjie Wang

**Affiliations:** 1 Department of Medical Oncology, Xiang’an Hospital of Xiamen University, School of Medicine, Xiamen University, Xiamen 361100, China; 2 Department of Medical Oncology, Xiamen Key Laboratory of Antitumor Drug Transformation Research, The First Affiliated Hospital of Xiamen University, School of Medicine, Xiamen University, Xiamen 361026, China; 3 Department of Thyroid and Breast Surgery, Xiamen Humanity Hospital Fujian Medical University, Xiamen 361006, China

**Keywords:** breast cancer, cell cycle, TPX2, immune infiltration

## Abstract

Background: This study aims to identify the essential cell cycle-related genes associated with prognosis in breast cancer (BRCA), and to verify the relationship between the central gene and immune infiltration, so as to provide detailed and comprehensive information for the treatment of BRCA.

Materials and Methods: Gene expression profiles (GSE10780, GSE21422, GSE61304) and the Cancer Genome Atlas (TCGA) BRCA data were used to identify differentially expressed genes (DEGs) and further functional enrichment analysis. STRING and Cytoscape were employed for the protein-protein interaction (PPI) network construction. TPX2 was viewed as the crucial prognostic gene by the Survival and Cox analysis. Furthermore, the connection between TPX2 expression and immune infiltrating cells and immune checkpoints in BRCA was also performed by the TIMER online database and R software.

Results: A total of 18 cell cycle-related DEGs were identified in this study. Subsequently, an intersection analysis based on TCGA-BRCA prognostic genes and the above DEGs identified three genes (TPX2, UBE2C, CCNE2) as crucial prognostic candidate biomarkers. Moreover, we also demonstrated that TPX2 is closely associated with immune infiltration in BRCA and a positive relation between TPX2 and PD-L1 expression was firstly detected.

Conclusions: These results revealed that TPX2 is a potential prognostic biomarker and closely correlated with immune infiltration in BRCA, which could provide powerful and efficient strategies for breast cancer immunotherapy.

## INTRODUCTION

Breast cancer is considered as one of the most common malignancies among women, with poor prognosis, high rates of local recurrence and distant metastasis [1, 2]. In recent years, immunotherapy, the main treatment methods for breast cancer, has also made significant progress in the field of breast cancer clinical therapy [3, 4]. Immune checkpoint inhibitors, such as programmed death ligand-1 (PD-L1) inhibitors and programmed death-1 (PD-1), showed the promising efforts in malignant breast cancer [5]. However, the clinical effect of PD-1/PD-L1 inhibitor monotherapy in the treatment of immunogenically “cold” tumors like breast cancer is not ideal, and it is often used in combination with chemoradiotherapy [6]. Previous studies have demonstrated that significant classic biomarkers related to targeted chemotherapy such as ER, PR and HER2 were involved in the tumor immune microenvironment and regulate immune response of BRCA patients. Nevertheless, low overall response rate and high acquired resistance rate still happen to most of BRCA patients treated with immunotherapy [7]. Therefore, the exploitation of novel immune-related targets in breast cancer are critically needed.

Another essential molecular characteristic that affects breast cancer progression is the dysfunction of cell cycle, which often involves the abnormal expression of cell cycle-related genes (CCRGs). For example, increased Cyclin D1 expression could result in the phosphorylation of the suppressor gene Rb, thereby leading to proliferation and metastasis of breast cancer [8]; p21-activated kinases-dependent upregulation of cyclin D1 is essential for G1/S transition, also can regulate breast cancer migration [9]. Recent studies have focused on the direction of how to use cell cycle checkpoints effectively targeted immunotherapy. It has been demonstrated that P21 recruited macrophages under stress by promoting the release of CXCL14 and other chemokines, and subsequently exerts an immune surveillance role *in vivo* [10]. Therefore, mining new cell cycle checkpoints and establishing immune network connections is becoming increasingly popular theme.

The targeting protein for Xenopus kinesin-like protein 2 (TPX2) is a microtubule-associated protein responsible for mitotic spindle formation, thereby influencing the biological process of cell cycle [11–13]. A previous study further revealed that peptides derived from TPX2 could be recognized by human cytolytic T lymphocytes (CTLs) and triggered a series of immune responses in hepatocellular cancer (HCC) [14, 15]. Meanwhile, another research showed that TPX2 depletion in HCC-infiltrating CD8 + T cells restricted the antitumor activity of CD8 + T cells and attenuated the efficacy with anti-PD-1 therapy [16]. Besides, high TPX2 expression was related to poor prognosis in various tumors, including pancreatic cancer, breast cancer, lung cancer, etc. [17–20]. These studies demonstrate that TPX2 could be a molecular prognostic and immunogenicity biomarker. However, the role and underlying functions of TPX2 in the progression and immunology of breast cancer is still vague.

Previous studies have proved that PD-L1 expression levels in breast cancer was positively correlated with the immune evasion, having a worse endpoint. Whether PD-L1 inhibitors effectively play a therapeutic role is closely linked to the PD-L1 expression status in breast cancer patients [21]. Thus, this study attempted to explore the functional effect of TPX2 on modulating PD-L1 expression in breast cancer. Moreover, the interaction of TPX2 with other immune components containing immune cells and other checkpoints was also analyzed in this study. These findings not only cast light on the crucial role of TPX2 in breast cancer and provide a new strategy on immune checkpoint blockade therapy, but also strength the link between cell cycle and immunity.

## MATERIALS AND METHODS

### Data collection and preprocessing

The raw data for GSE10780, GSE21422 and GSE61304 were extracted from the GEO database: GSE10780 included 42 tumor tissues and 143 normal tissues, dataset GSE21422 included 14 cancer tissues and 5 normal tissues, and dataset GSE61304 included 58 cancer tissues and 4 normal tissues. All GEO datasets were merged into the GPL570 platform. Meanwhile, the clinical data and expression profiles of BRCA cases were retrieved from TCGA database, reference CCRGs set were extracted from MSigDB2, and “GO_CELL_CYCLE” module was also selected to screen optimal cell cycle-related genes. Furthermore, additional GEO datasets (GSE110686, GSE176078, GSE148673) and EMTAB8107 dataset were downloaded for analyzing TPX2 expression level across different cell types.

### Identification of differentially expressed genes and functional annotation

“Limma” package was used for the identification of DEGs with the screening criteria of adjusted value of p<0.001 and | log fold change (FC)| >1.5. Venn diagram and volcano plot were depicted via Ggplot2 and Venn diagram R packages, respectively, for the visualization of the identified DEGs. Furthermore, gene Ontology (GO) analysis for the DEGs were explored using “ClusterProfiler” package, and p-value < 0.05 was considered statistically significant. STRING database and Cytoscape 3.7.2 software were used to construct PPI network for further exploitation of the core gene groups with the most interactions [22, 23].

### Identification of prognosis-related DEGs and survival analysis

The dataset “TCGA-BRCA” with abundant clinical information from huge sample size was used to screen significant DEGs correlated with survival outcome, and Venn diagram package was performed for illustrating the prognosis-related DEGs. The Kaplan-Meier method with the log-rank test was employed for analyzing the survival endpoint of these genes in the study. Multivariate and univariate Cox regression analyses including specific indicators, age, pathological stage, TNM stage, TPX2 expression, were performed in the study, and the nomogram analysis was depicted via “RMS” package.

### Correlation analysis of clinicopathological features

In this study, we extracted TCGA-BRCA data with clinical parameter information to analyze the relationship between TPX2 expression and clinical subgroup variate, and Kruskal-Wallis test method was employed for the measurement of the statistical difference and Ggplot2 was used to visualize this result.

### Analysis of TPX2-related genes and proteins

The top 100 TPX2-correlated genes based on TCGA-BRCA cohort was examined by the section “Similar Genes Detection” of GEPIA2 tool [24]. Further, the top 5 genes were adopted to perform Pearson correlation analysis with TPX2. Moreover, using the “Gene_Corr” module of TIMER2, a heatmap of the association between these 5 genes and TPX2 in pan-cancer was applied for the Spearman’s correlation test. Additionally, protein-protein interactions with TPX2 were performed using the STRING tool. An estimated 48 experimental proteins were identified as the TPX2-interacted proteins. Then, Venn diagram was used for the intersection analysis of TPX2-correlated and TPX2-interacted genes. Meanwhile, “clusterProfiler” was applied to testify the biological processes of the TPX2-related genes.

### Correlation between TPX2 expression and immune infiltration

To further explore the role of TPX2 expression in immune microenvironment of BRCA, we downloaded the transcriptome data of 1082 breast cancer patients with normal samples excluded from the TCGA database. CIBERSORT algorithm was employed to calculate infiltration abundance ratio of 22 kinds of immune cells in breast cancer tissue. “Ggcorr” package was used to evaluate the correlation between immune-infiltrating cells, and the relationship between the low and high expression of TPX2 and 22 kinds of breast cancer immune cells. Furthermore, TISIDB tool was explored to illustrate the association between tumor-infiltrating lymphocytes (TILs), TPX2 expression and immunomodulators using the Spearman test [25]. TIMER database was used for evaluating the relationship with TPX2 and the markers of TILs [26]. Besides, the X-ray crystal structures of TRX2 (6BJC) and (3BIK) were retrieved from the Protein Data Bank, and AutoDockTools-1.5.7 was applied for the molecular docking of TPX2 and PD-L1 [27]. The water molecules were manually eliminated from the protein and the polar hydrogen was added. Docking Web Server (GRAMM) was used for protein-protein docking and PyMOL software was explored for the visualization [28].

### Cell culture and reagents

All human breast cancer cell lines MCF-7, BT-474, T-47D, MDA-MB-453, MDA-MB-231, SUM-159PT and normal breast cell line MCF-10A were purchased from the American Type Tissue Collection (ATCC, USA) and cultured as stated in the manufacturer’s instructions [29]. These cell strains were cultured in an incubator with a criteria of 5% CO2 humidified atmosphere and 37° C constant temperature. Cell culture-mediums were commercially purchased from the Procell Life Science and Technology Co., Ltd. (China).

### Plasmid DNA transfections

The Flag-tagged TPX2 was cloned into the pLenti-CMV-blast vector, then, plasmid DNA was transfected into MDA-MB-231 cells using OPTI-MEM and Lipofectamine 3000 (Invitrogen, USA) reagents according to the manufacturer’s instructions [30], and transfected cells were collected at 48 h after transfections.

### qRT-PCR analysis

Total RNA was extracted by cells using Trizol reagent and cDNA reverse transcription was applied for a SPARKscript II RT kit (Shandong Sparkjade Biotechnology Co., Ltd., China). The Hieff®qPCR SYBR Green Master Mix (11201ES08) was used for the subsequent qRT-PCR process (Yeasen Biotechnology (Shanghai) Co., Ltd., China). The 2^ΔΔcT^ method was used to ascertain the expression of the target genes. All experiments were conducted in triplicate. The primers used were as follows:

TPX2 forward, 5′- AGACTGACAGAAGAGGTGCT -3′,

TPX2 reverse, 5′- GATGACGGTGTTTGGACGAG -3′;

β-Actin forward, 5′- CGTGCGTGACATTAAGGAGAAG -3′,

β-Actin reverse, 5′- GGAAGGAAGGCTGGAAGAGTG -3′.

### Flow cytometry

Flow cytometry was used for the detection of cell surface PD-L1 expression in this study. Cells were digested and washed three times with cold PBS after centrifugation, and then incubated with CD274-PE (329705; 1:50; Biolegend, USA) or Mouse IgG2b Isotype Control-PE (400313; 1:50; Biolegend) for 30 min on ice. Finally, cells were washed via cold PBS again and detected by flow cytometry.

### Immunohistochemistry (IHC) assays and scoring of the staining

The tissue microarrays (Bioaitech, F175Br01, China) used in the study included a total of 175 samples, containing 145 breast cancer tissue samples with various grades, stages and 30 matched normal samples. Anti-TPX2 (ABclonal, A18327, China) performed at a concentration of 1:250, and the primary antibody against PD-L1 (Abcam, ab205921, USA) was diluted 1:200 in the process of immunohistochemistry assays. All stained slides were scanned on AxioScan Z1 (Zeiss, Germany) software. Computerized image analysis was performed by Aipathwell and final scoring results were mainly based on the positive cell density (number/mm^2^) [31]. The correlation analysis was executed by Student’s t-test and p < 0.05 was considered statistically significant.

### Availability of data and material

The data used in this study are available from the corresponding authors.

### Consent for publication

All authors gave permission for this study to be published.

## RESULTS

### Identification of DEGs in breast cancer and the enrichment of these genes

**Figure 1:**
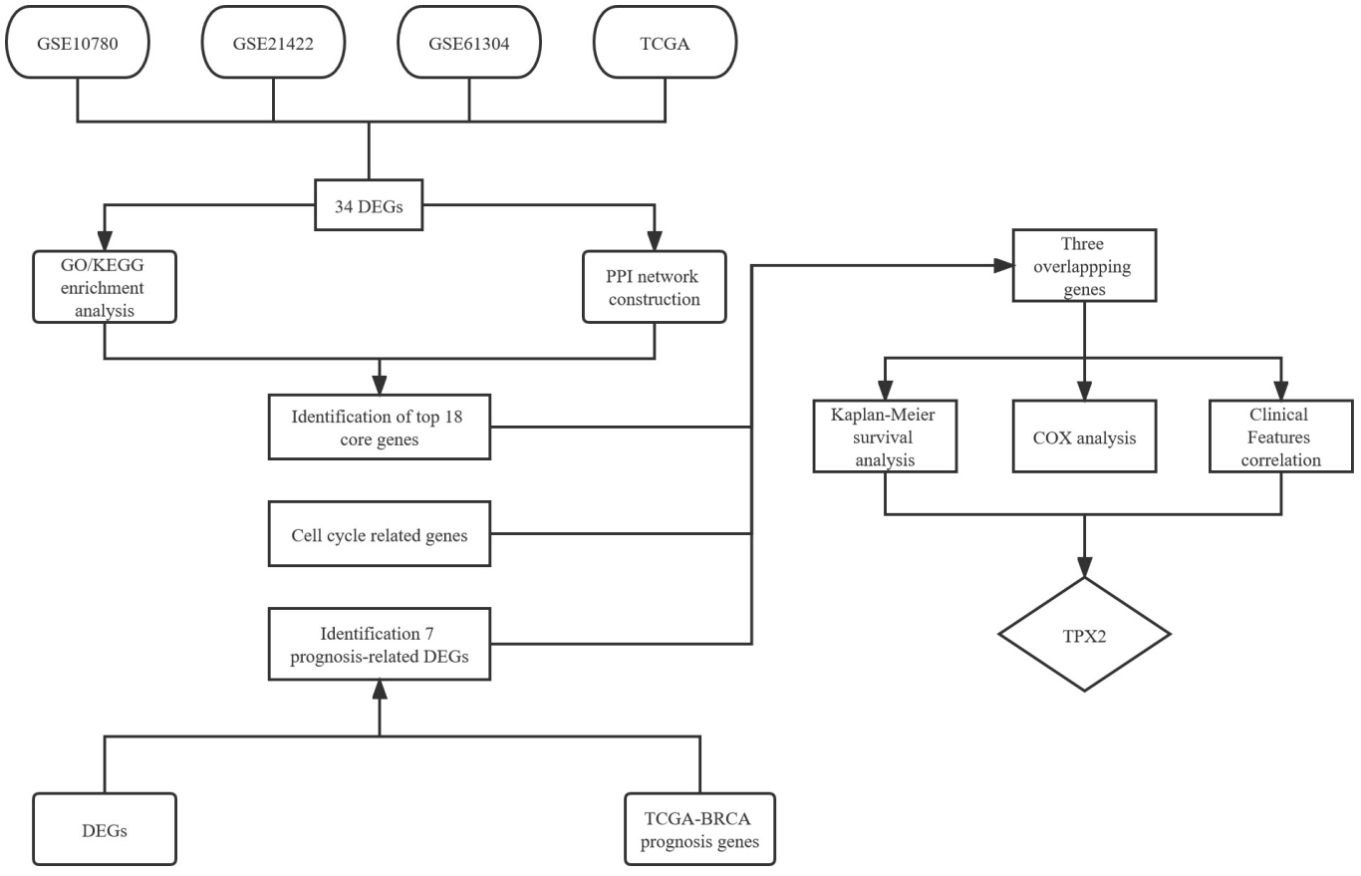
Flow chart of the screened and validated process.

**Figure 2:**
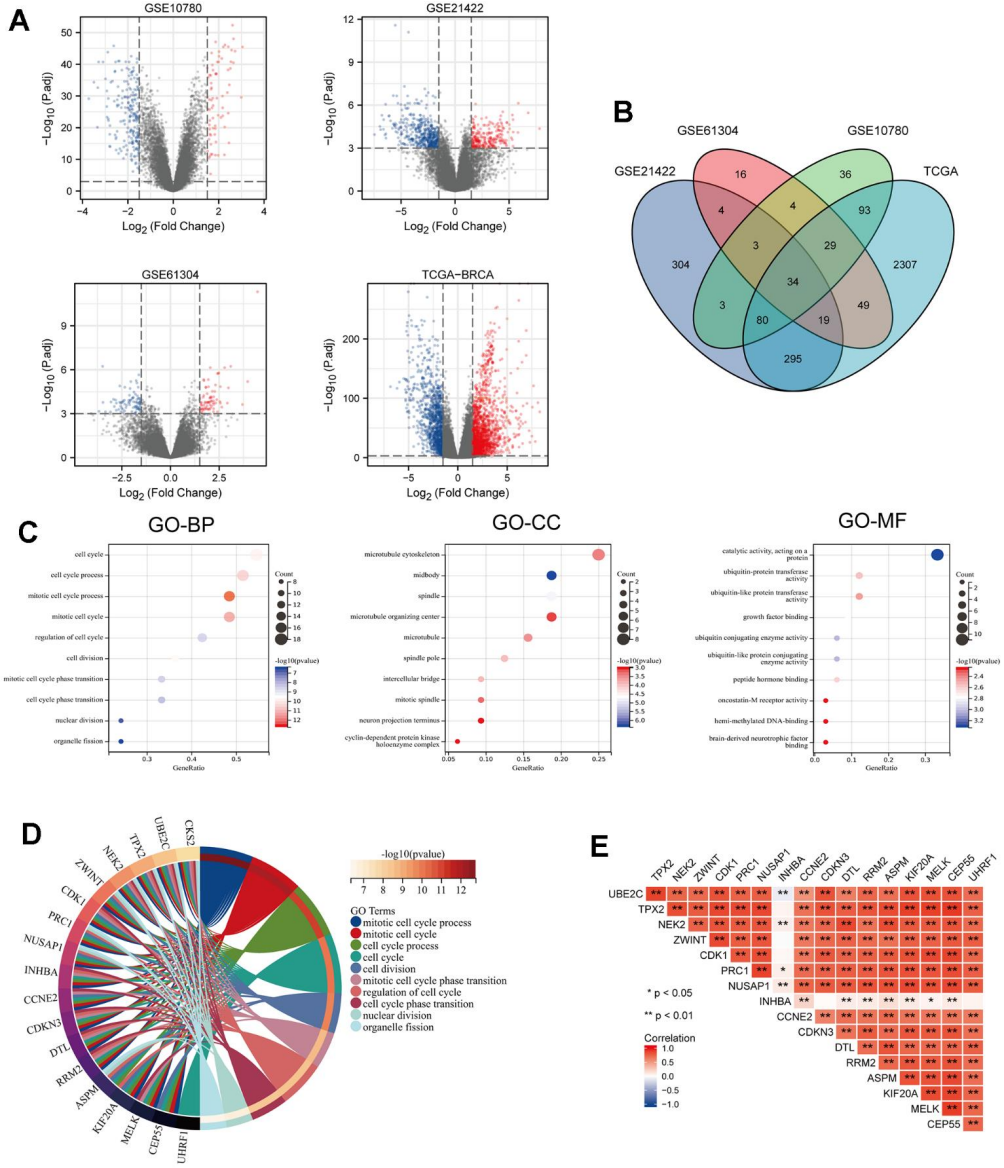
**Identification of DEGs from 3 GEO datasets and TCGA dataset of breast cancer (BRCA) and GO enrichment analysis of consistent DEGs of the datasets.** (**A**) Volcano maps of genes detected in BRCA datasets. Red and blue represent up- and downregulated genes, respectively. (**B**) Venn diagram showing overlapped part of DEGs in four BRCA datasets. (**C**) GO enrichment analysis of the overlapped part of the DEGs. (**D**) Circle enrich plot. GO enrichment analysis for 34 DEGs, terms with p < 0.05 was believed to be enriched significantly. (**E**) The association of these 18 candidate genes in breast cancer, *P < 0.05, **P < 0.01.

In this study (Figure 1), three microarray data sets (GSE10780, GSE21422, GSE61304) and the TCGA-BRCA dataset were downloaded and processed via “limma” R package with the criteria of the | logFC| > 1.5 and adjusted *p* < 0.001, and all DEGs were displayed in volcano maps (Figure 2A). The overlapping DEGs among these four datasets included 34 genes as shown in the Venn diagram (Figure 2B), and their expression profile was illustrated in a heatmap (Supplementary Figure 1A). To clarify the major functions of these DEGs, we further performed the GO functional annotation and the results showed DEGs were remarkably related to cell cycle, mitotic cell cycle process, microtubule cytoskeleton, midbody, spindle (Figure 2C). Then, the STRING database was used for predicting the potential relationships among these DEGs, as illustrated in Supplementary Figure 1B, and the PPI network construction via Cytoscape software was shown in Supplementary Figure 1C, including 18 nodes and 300 edges. According to degree ≥ 30, 18 core genes including CKS2, UBE2C, TPX2, NEK2, ZWINT, CDK1, PRC1, NUSAP1, INHBA, CCNE2, CDKN3, DTL, RRM2, ASPM, KIF20A, MELK, CEP55, UHRF1 were identified. Meanwhile, we selected the highest degree of DEGs for functional analysis in Supplementary Table 1. We re-displayed the GO enrichment results by R package “ClusterProfiler”, revealing that DEGs were mostly enriched in cell cycle related pathway (Figure 2D). Finally, the correlation of these 18 crucial genes in BRCA samples was identified in the study, suggesting that most of them had a positive relationship (Figure 2E).

### Identification cell cycle-related signature in BRCA

**Figure 3:**
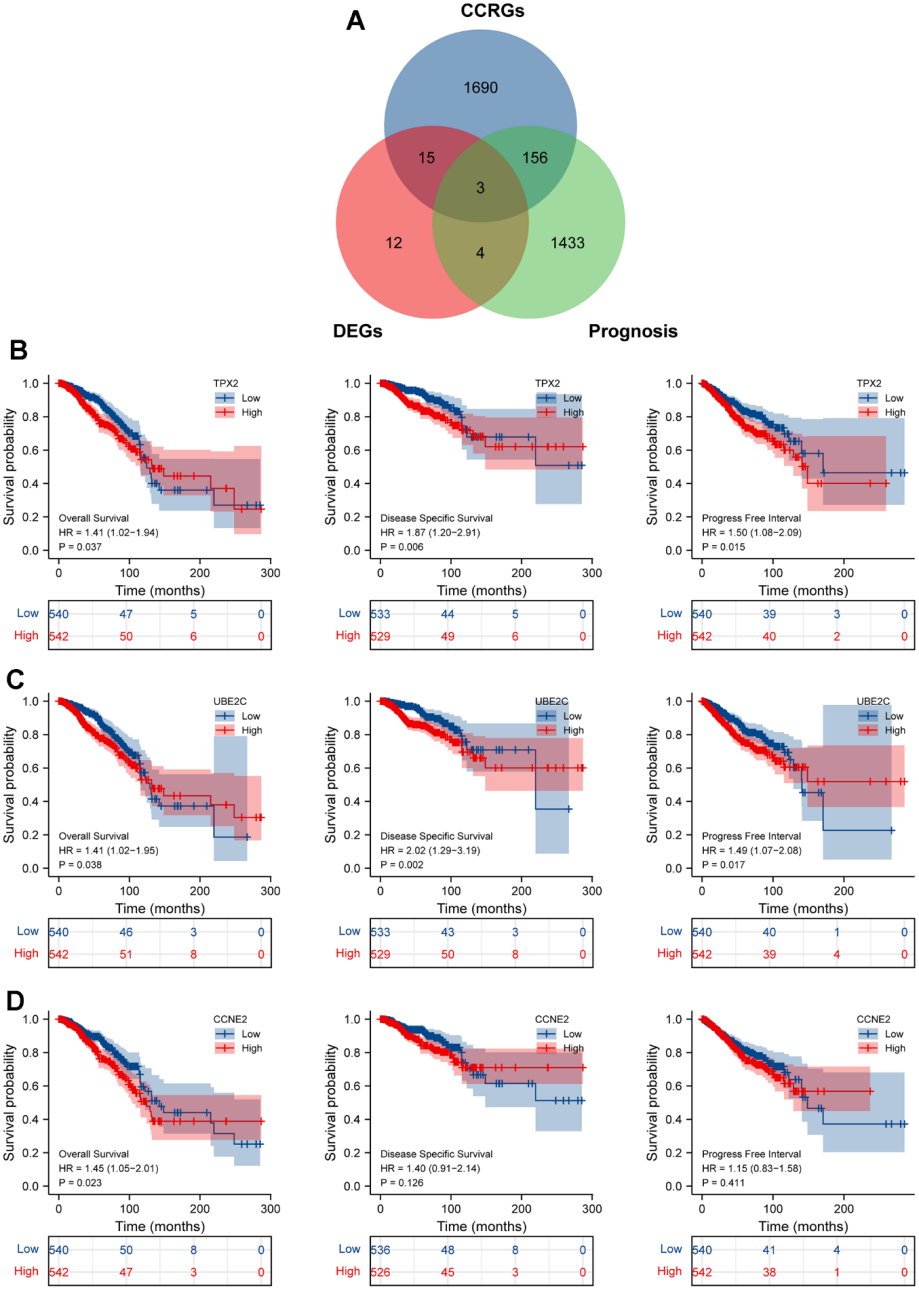
**Identification cell cycle-related DEGs.** (**A**) An intersection analysis of the TGCA-BRCA prognostic molecules and cell cycle-related DEGs. (**B**–**D**) The survival analysis of TPX2/UBE2C/CCNE2 with the indictor, overall survival (OS), disease specific survival (DSS), progress free interval (PFI), respectively. P <0.05 was considered significant.

Based on the GO enrichment results, we downloaded the CCRGs set from the MSigDB2 database and further single out the above 18 genes as the cell cycle-related DEGs. Then, we explored the prognostic significance of these cell cycle-related DEGs by the intersection of the TCGA-prognosis cohort, and suggested that only TPX2, UBE2C and CCNE2 were significantly associated with overall survival (Figure 3A). We re-analyzed the relationship between survival indicators (OS, DSS and PFI) and the expression of these three selected key genes. The results found that TPX2 and UBE2C expression were significantly related to these three survival indicators, suggesting they could be potential prognosis biomarkers in BRCA patients (Figure 3B–3D).

**Figure 4:**
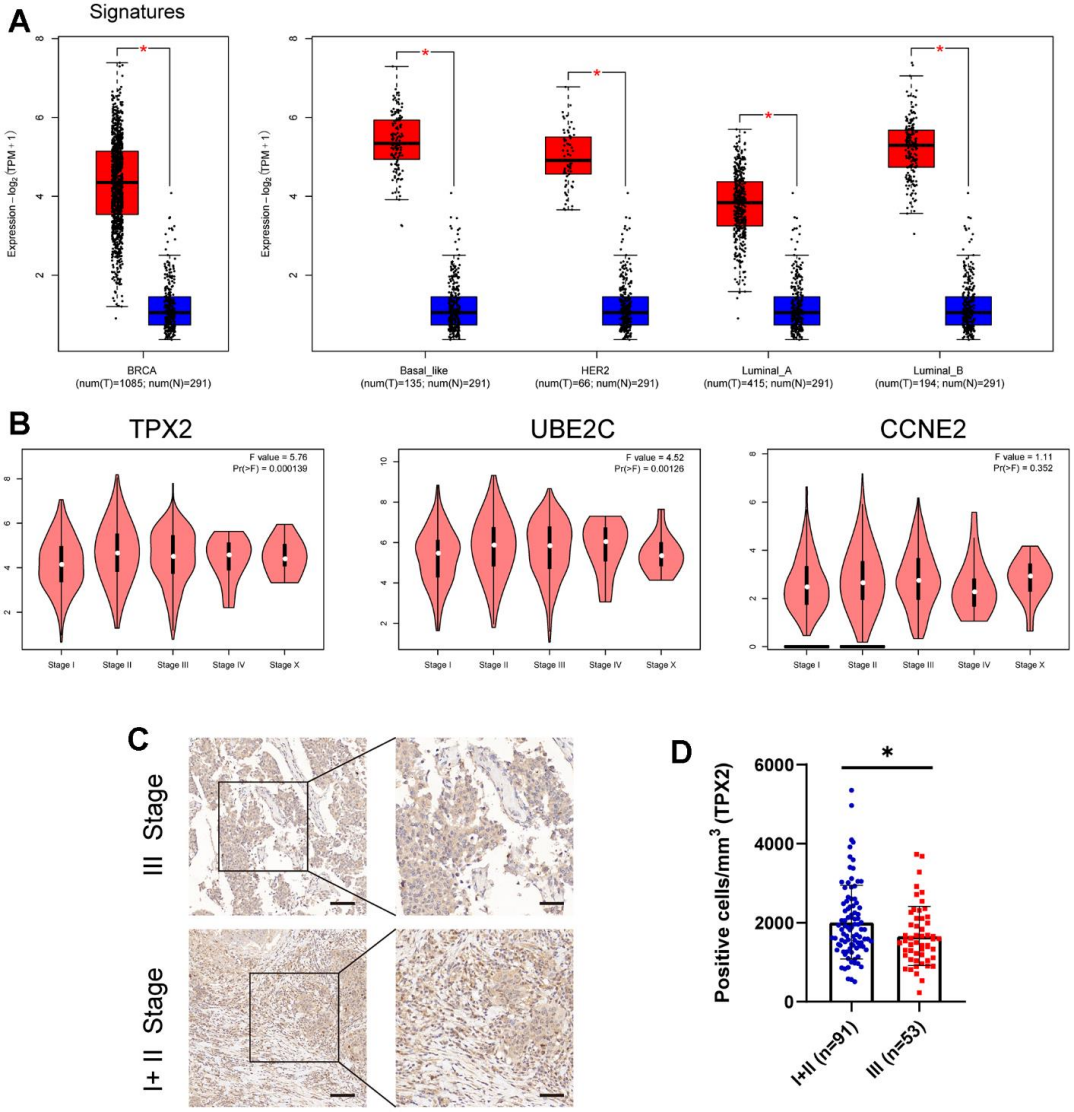
(**A**) The expression level of these three candidate cell cycle-related DEGs in BC or four BC subtypes (Basal-like; HER2; Luminal-A; Luminal-B) and normal patients. (**B**) Correlations between TPX2/UBE2C/CCNE2 expression and tumor stage in BRCA patients. P<0.05 is considered as difference. (**C**, **D**) Immunohistochemistry detecting the protein level of TPX2 in various stages. *P < 0.05. Scar bar: 100 μm.

Three CCRG signatures were processed as a CCRG set, and association analysis of BRCA subtypes and stages between normal samples and BRCA was examined using the GEPIA2 tool. As illustrated in Figure 4A, the expression level of the CCRG set in BRCA patients was higher than normal group. Similarly, analysis of the BRCA subtypes also showed the same result in the four BC subtypes (Basel-like, HER2, Luminal-A and Luminal-B) compared with the normal group. Addedly, we further examined the association between these three genes expression and clinical stage, and found that the differential expression of TPX2 is most significant in different stages of BRCA (Figure 4B). Subsequently, we verified that the expression of TPX2 in I+ II stage was significantly higher than that in III stage by the Immunohistochemical test (Figure 4C, 4D). These results illustrated TPX2 may be an efficient and promising biomarker for evaluating the progression of BRCA.

### The connection between expression level of TPX2 and survival and clinicopathologic variables

**Figure 5:**
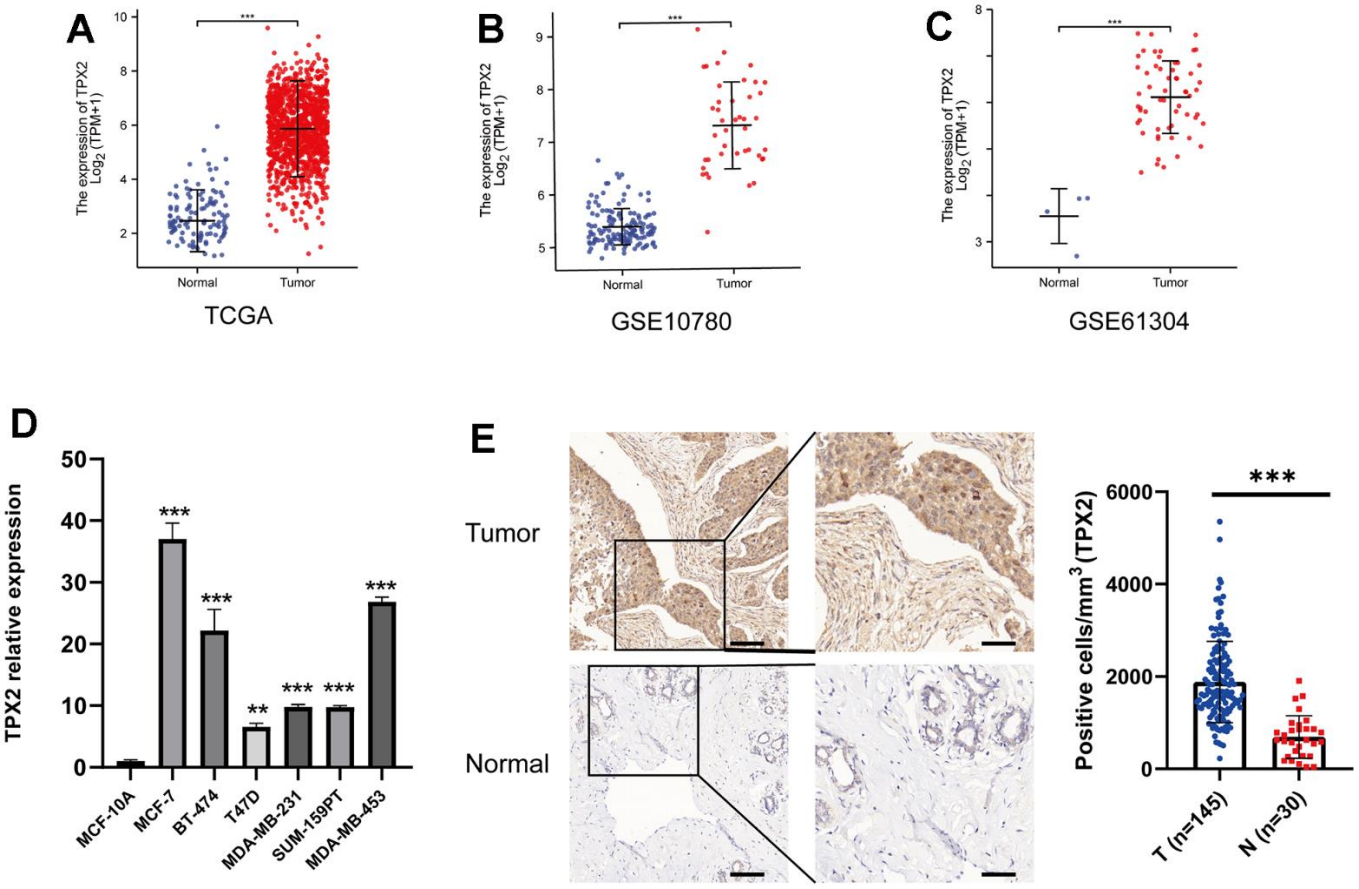
**Expression profile and survival situation of TPX2 in BRCA.** (**A**–**C**) Differential expression levels of TPX2 in BRCA from TCGA and GEO database. (**D**) The mRNA expression of TPX2 in breast cancer cell lines. (**E**) The differential protein level of TPX2 in breast tumor and normal tissues. **P < 0.01, ***P < 0.001.

Aiming to conduct a comprehensive analysis on the role of TPX2 in BRCA, we compared the expression of TPX2 in tumor and matched normal samples by GEO and TCGA datasets, suggesting that TPX2 was overexpressed in breast cancer (Figure 5A–5C). Moreover, we compared the gene expression difference of TPX2 in breast cancer cells and normal breast cell and found that TPX2 was higher expressed in breast cancer (Figure 5D). The IHC experiment detecting BRCA and normal tissues also demonstrated this similar result (Figure 5E). Besides, univariate and multivariate Cox analyses also demonstrated that TPX2 acted as a detrimental prognostic factor in BRCA (Table 1). Moreover, we extracted additional GEO datasets for prognostic analysis of TPX2 using PrognoScan and Km-plotter website tool, which was a revalidation of the previous conclusion (Supplementary Figure 2 and Supplementary Table 2).

**Table 1:** Univariate and multivariate Cox analyses of the correlation of TPX2 expression with overall survival (OS) among breast cancer patients.

**Characteristics**	**Univariate analysis**			**Multivariate analysis**	
	**Hazard ratio (95% CI)**	**P-value**		**Hazard ratio (95% CI)**	**P-value**
Age (<=60 vs >60)	2.020 (1.465-2.784)	**<0.001**		2.141 (1.484-3.089)	**<0.001**
Pathologic stage (I+ II vs III+ IV)	2.391 (1.703-3.355)	**<0.001**		1.791 (1.040-3.083)	**0.035**
T stage (T1+T2 vs T3+T4)	1.608 (1.110-2.329)	**0.012**		0.948 (0.565-1.591)	0.841
N stage (N0 vs N1+N2+N3)	2.239 (1.567-3.199)	**<0.001**		1.753 (1.126-2.730)	**0.013**
M stage (M0 vs M1)	4.254 (2.468-7.334)	**<0.001**		2.122 (1.051-4.284)	**0.036**
TPX2: High vs Low	1.409 (1.022-1.944)	**0.037**		1.588 (1.112-2.266)	**0.011**

The value in bold indicates that p is less than 0.05, which is meaningful. HR, hazard ratio; CI, confidence interval.

**Figure 6:**
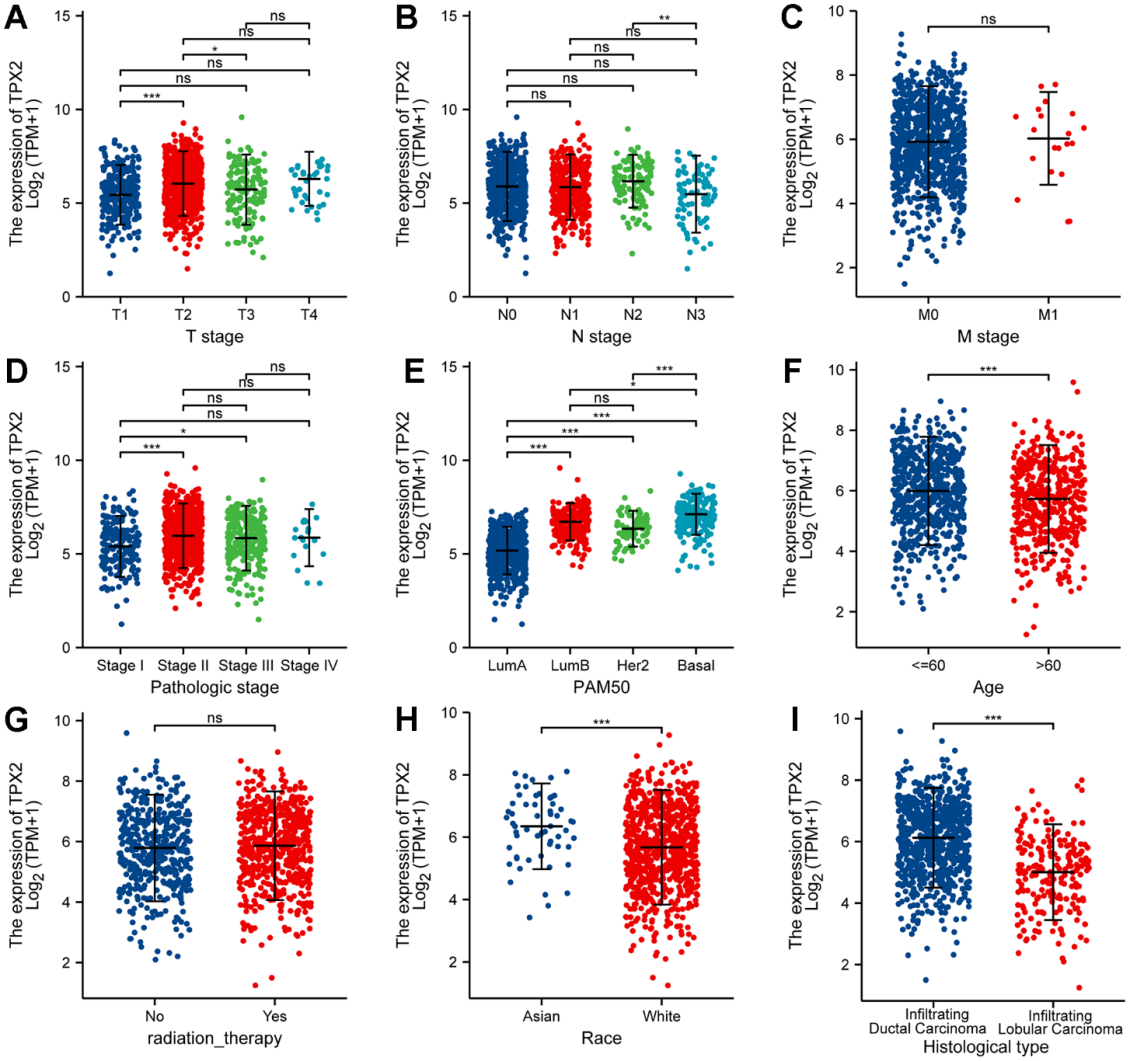
Correlation between TPX2 expression and the clinicopathological features of breast cancer patients for (**A**) T stage, (**B**) N stage, (**C**) M stage, (**D**) pathologic stage, (**E**) PAM50, (**F**) Age, (**G**) radiation therapy, (**H**) race and (**I**) histologic type. *P < 0.05, **P < 0.01, ***P < 0.001.

As is depicted in Figure 6, high expression of TPX2 was significantly associated with T stage (T1 vs T2, T2 vs T3), N stage (N2 vs N3), pathologic stage (stage I vs stage II and III), PAM50 (LumA vs LumB, Her2 and Basal), age (<=60 vs >60), race (Asian vs White) and histological type (Infiltrating Ductal Carcinoma vs Infiltrating Lobular Carcinoma). Additionally, we analyzed the association between TPX2 expression and clinical features using the tissue microarray, and discovered that TPX2 expression had a close tie with the stages and grades of BRCA (Supplementary Table 3).

### Association between TPX2 mutation and survival in breast cancer

To further explore the correlation between TPX2 expression and the landscape gene mutations, we downloaded the mRNA expression data of TPX2 from the TCGA database and clarified into the high TPX2 group including 498 samples and low TPX2 group including 454 samples based on the median value of TPX2 expression level. The results demonstrated that more mutations were detected in high TPX2 expression group, and high frequency of mutations in TP53, PIK3CA, TTN, MUC16, HMCN1, RYR2, USH2A, FLG was discovered in BRCA with high TPX2 expression. Meanwhile, mutations in PIK3CA, TTN, TP53, CDH1, MUC16, MAP3K1 were enriched in samples with low TPX2 expression (Supplementary Figure 3A, 3B). Supplementary Figure 3B indicated that TPX2 has missense mutation, deletion and splice (Supplementary Figure 3C). Besides, we investigated that the CNV distribution of TPX2 and analyzed the relation with its mRNA expression, and found that the CNV level was positive with TPX2 gene expression, and TPX2 deletion had a worse prognosis than TPX2-wild and TPX2 amplified (Supplementary Figure 3D–3G). Additionally, we also examined a methylation prediction of TPX2 and its methylation was negative with its mRNA expression, and also found to significantly alter the prognosis of breast cancer patients (Supplementary Figure 4). These results suggested TPX2 mutation could exert an important influence on the survival of breast cancer patients.

### Association between TPX2 expression and immune infiltration in breast cancer

**Figure 7:**
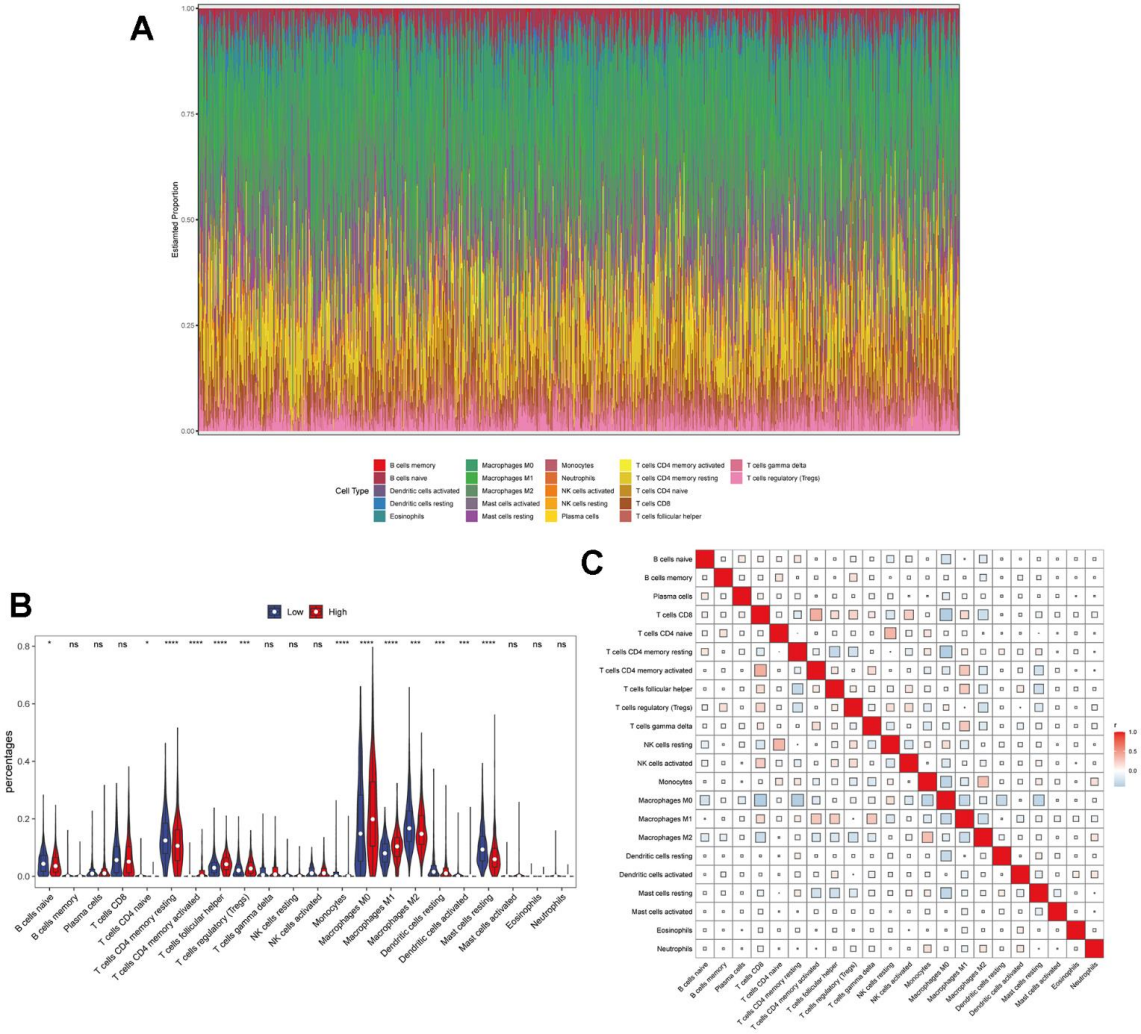
**The relationship between TPX2 expression and tumor-infiltrating immune cells.** (**A**) Stacked bar chart shows distribution of 22 immune cells in each sample. (**B**) Violin plot displays the differentially infiltrated immune cells between TPX2-High group and TPX2-Low group. Red color represents TPX2-High group, and blue color represents TPX2-Low group. (**C**) Correlation matrix of immune cell proportions. The red color represents positive correlation and the blue color represents negative correlation.

Previous studies have revealed that TPX2 was closely involved in the immune process of some types of cancer, including lung adenocarcinoma, papillary renal cell carcinoma and hepatocellular carcinoma [32, 33]. However, the connection between TPX2 expression and immune infiltration of BRCA has not clearly elucidated. Thus, we downloaded transcriptome data of 1082 BRCA patients from the TCGA database for immune infiltration analysis. As is shown in Figure 7A, 7C, the results displayed the composition of 22 immune cell types and the mutual relationship with these immune-infiltrating cells. More importantly, high expression level of TPX2 has showed to be connected to specific immune cells, such as dendritic cells, monocytes, mast cells, CD4 T cells and macrophages, which suggested that TPX2 was closely implicated in the immune response and might regulate the immunotherapy of BRCA (Figure 7B). Through single-cell transcription analysis, we found that TPX2 was mainly distributed in proliferating T cells from GSE110686, GSE176078, EMTAB8017 and GSE148673 database (Supplementary Figure 5). Moreover, we analyzed the relationship between TPX2 expression and immune marker sets of BRCA using TIMER database, and discovered that TPX2 expression was associated with most immune markers (Supplementary Table 4). These results suggested that TPX2 could be an essential and powerful immunotherapy-relevant biomarker for controlling the condition of BRCA patients.

### The relationship between TPX2 and PD-L1 in breast cancer

**Figure 8:**
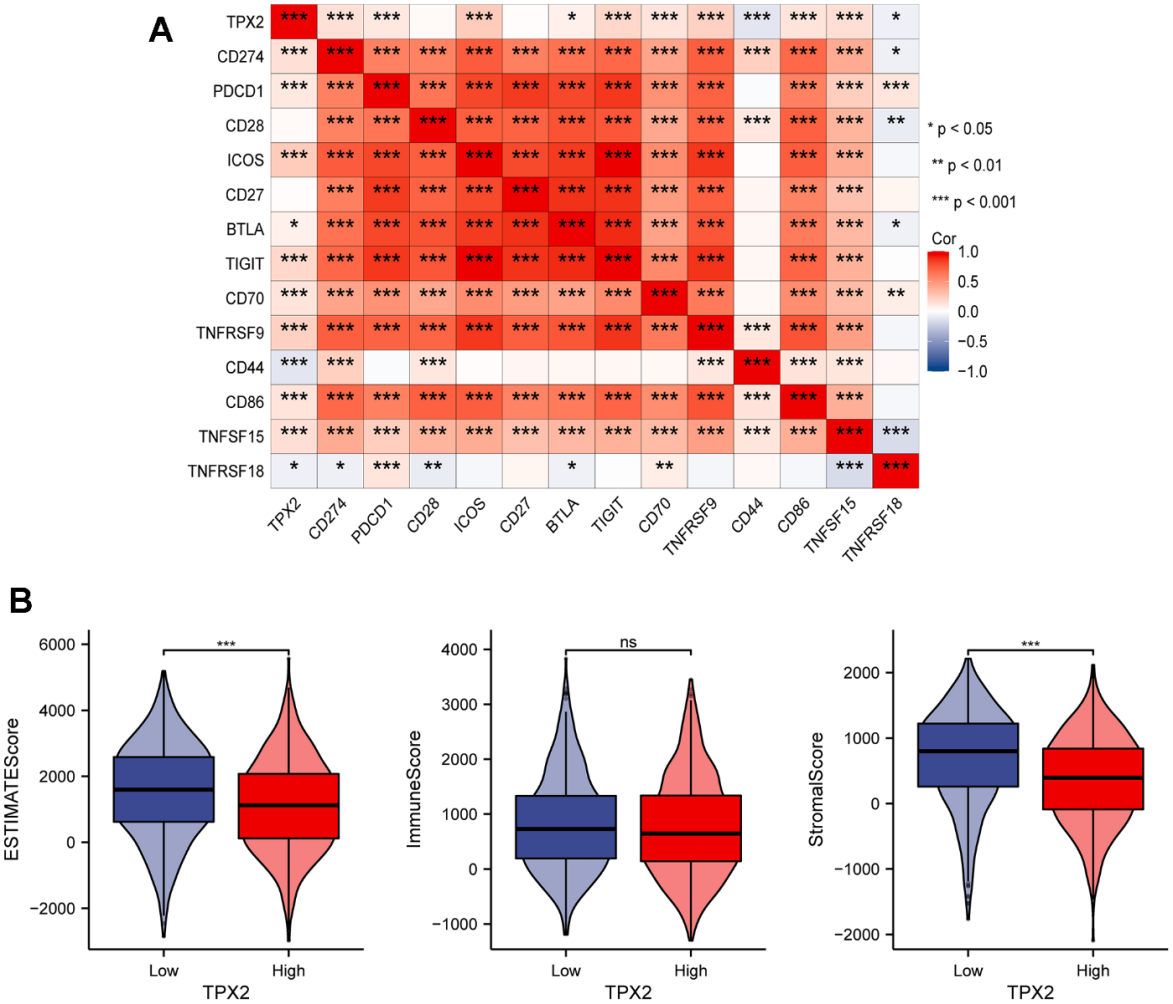
(**A**) Correlation analysis between TPX2 expression and representative immune checkpoints from TCGA database. (**B**) The relationship between TPX2 expression and immune microenvironment. *P < 0.05, **P < 0.01, ***P < 0.001.

The immune system controls its overreaction through the regulation of multiple immune checkpoints, which helps prevent the immune system from damaging healthy tissue. Interestingly, some cancer cells could utilize specific checkpoints, such as PD-L1 and PD-1, to evade immune system surveillance [34]. To further comprehend the significance of TPX2 in immune response of BRCA, we selected 13 representative immune checkpoints (CD274, PDCD1, CD28, ICOS, CD27, BTLA, TIGIT, CD70, TNFRSF9, CD44, CD86, TNFSF15, TNFRSF18) to analyze their relation with TPX2 expression (Figure 8A). Besides, we also detected the relationship between TPX2 expression and immune microenvironment through analyzing the relevancy with three indicators (immune score, estimated score, and stromal score), and suggested that TPX2 could be a strong immune regulator of BRCA (Figure 8B).

**Figure 9:**
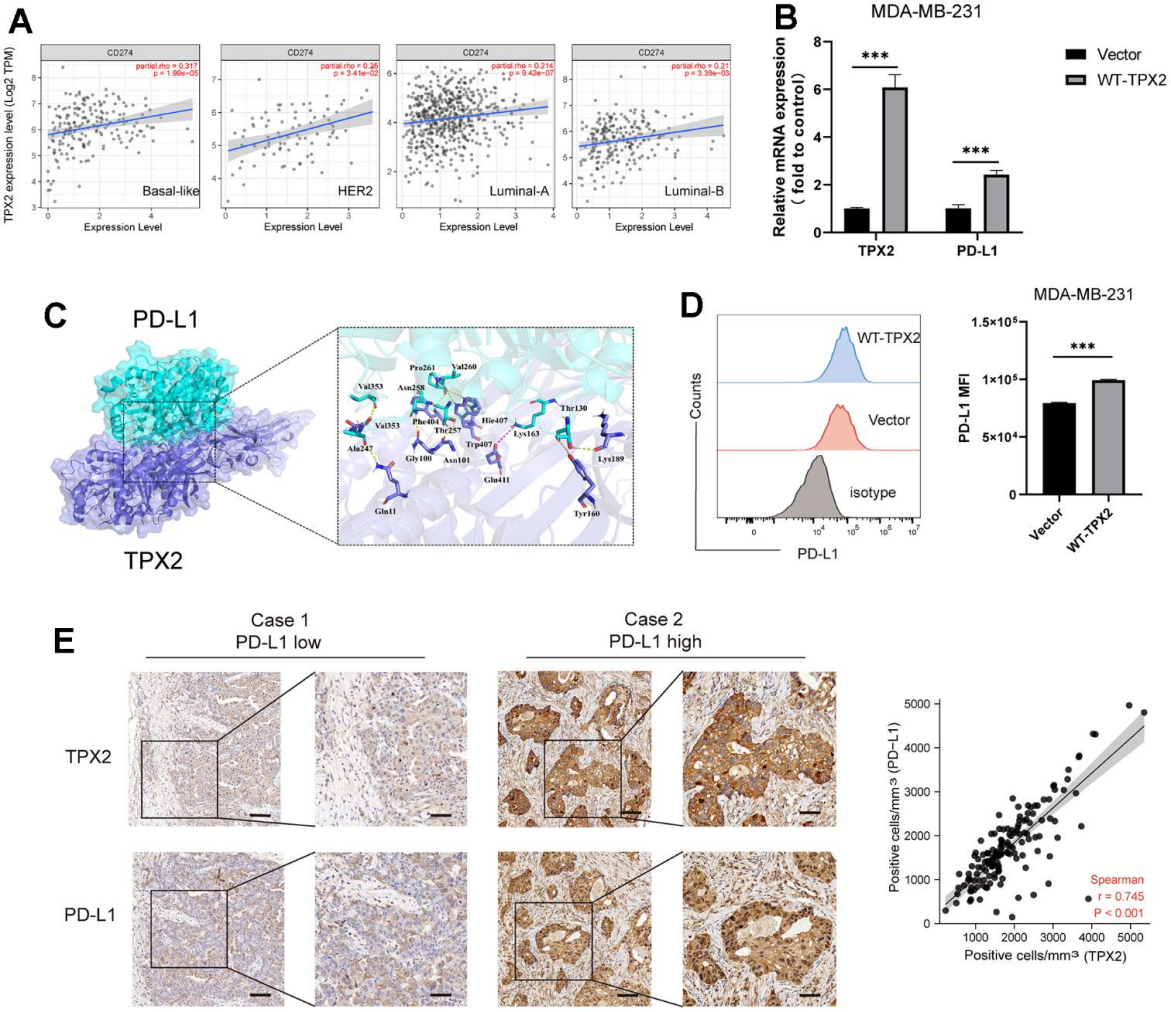
**The relationship between TPX2 and PD-L1 in BRCA.** (**A**) TIMER database illustrating the relationship between TPX2 and CD274 (PD-L1) in four BC subtypes. (**B**) RT-QPCR experiment illustrating the gene relationship between TPX2 and PD-L1. (**C**) Molecular docking simulating the amino acid interaction between TPX2 and PD-L1. (**D**) Flow cytometry illustrating the protein relationship between TXP2 and PD-L1. (**E**) Immunohistochemistry detecting the protein level of TPX2 and PD-L1 from tissue microarray. Scar bar: 100 μm. ***P < 0.001.

PD-L1 was usually highly expressed in BRCA patients, especially triple-negative breast cancer (TNBC) patients, and responsible for the poor prognosis. In this study, we analyzed the relationship between TPX2 and PD-L1 in four BRCA subtypes and revealed that TPX2 expression was positively correlated with PD-L1 (Figure 9A). Furthermore, we also investigated the expression patterns of TPX2 and CD274 in GSE176078 dataset and found that TPX2 was positive with CD274 on cytotoxic T cell, ductal cell, and macrophage cells, which is consistent with TCGA database results (Supplementary Figure 6). Then, we selected a representative breast cancer cell line MDA-MB-231 commonly used for research to testify the positive regulation between TPX2 and PD-L1 using flow cytometry and RT-QPCR methods (Figure 9B, 9D). The interaction simulation between TPX2 and PD-L1 was also performed in this study through molecular docking technique (Figure 9C), and showed that they form lots of hydrogen bonds, such as Gln11-Ala247, Gly100-Asn258, and Trp407-Val260 (Supplementary Table 5). Furthermore, the relationship between TPX2 and PD-L1 was also observed using immunohistochemistry and the results showed that TPX2 expression was positive with PD-L1 (Figure 9E). The high expression of PD-L1 in BRCA tumors have been demonstrated to trigger severe immune escape and promote tumor fast development. Furthermore, we also analyzed the relation between TPX2 expression and TIDE score, TMB and MSI, indicating that TPX2-low group has higher TIDE score compared to that of TPX2-high group, and the tumor mutation burden of TPX2-high group has higher than that of TPX2-low group (Supplementary Figure 7). Thus, therapies targeting TPX2 may revive anti-tumor immune responses.

### TPX2-related genes and protein–protein interactions

**Figure 10:**
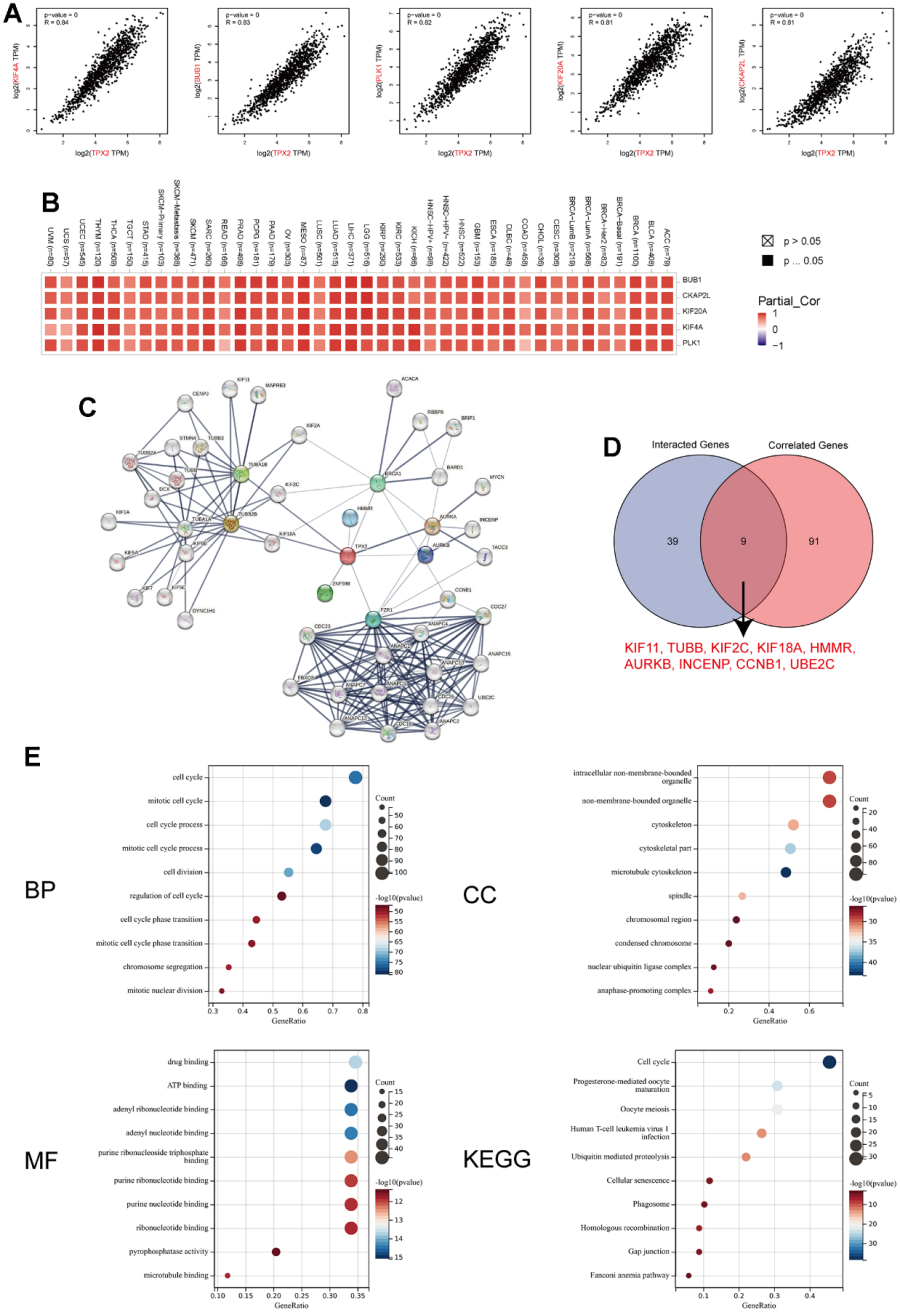
**TPX2-related gene network, protein–protein interactions and enrichment analysis.** (**A**) Top 5 TPX2-correlated genes in TCGA database, KIF4A, BUB1, PLK1, KIF20A and CKAP2L, Pearson’s correlation coefficients. (**B**) The heatmap revealing the correlation between TPX2 expression and these top 5 genes in various tumors. (**C**) PPI network construction of 48 experimentally verified TPX2-interacted proteins. (**D**) An intersection analysis of the TPX2-interacted and TPX2-correlated genes. (**E**) Enrichment analysis illustration based on the TPX2-interacted and TPX2-correlated genes. (BP: biological process; CC: cellular component; MF: molecular function; KEGG: Kyoto Encyclopedia of Genes and Genomes).

Then, we conducted the TPX2-related gene analysis and protein-protein interaction analysis to elucidate the mechanism of TPX2 in tumorigenesis. The top 100 TPX2-related genes were recognized in Supplementary Table 6, and top 5 most associated genes were illustrated in Figure 10A: Kinesin Family Member 4A (KIF4A, R=0.84), Budding Uninhibited By Benzimidazoles 1 (BUB1, R=0.83), Polo Like Kinase 1 (PLK1, R=0.82), Kinesin Family Member 20A (KIF20A, R=0.81), Cytoskeleton Associated Protein 2 Like (CKAP2L, R=0.81). Furthermore, the association between TPX2 expression and these top 5 genes in all tumor types from the TCGA database was also illustrated in the heatmap (Figure 10B). The protein–protein network analysis demonstrated a total of 48 proteins experimentally interacted with TPX2 in this study (Figure 10C). Subsequently, an intersection analysis based on the TPX2-correlated and TPX2-interacted genes was also performed in this study, and the results showed 9 common member genes: KIF11, TUBB, KIF2C, KIF18A, HMMR, AURKB, INCENP, CCNB1 and UBE2C (Figure 10D). The KEGG and GO enrichment analysis of the two combined gene datasets illustrated that TPX2-related genes were related to the biological process of cell cycle, intracellular non-membrane-bounded organelle, drug binding, ATP binding and others (Figure 10E). These results strongly indicated that TPX2 could regulate the process of tumor cell cycle and may further decided the fate of malignance tumors.

## DISCUSSION

Despite significant therapeutic strategy advances, breast cancer remains to be the main cause of cancer death in women. Breast cancer is a highly heterogeneous group of tumors due to genetic mutations and stem cell differentiation, and chemotherapy is still the primary treatment for BRCA [35, 36]. However, drug resistance and recurrence lead most breast cancer patients to poor clinical outcomes. Therefore, robust and promising biomarkers to accelerate clinical progress are urgently needed. In this study, we primarily integrated three breast carcinoma GEO (GSE10780, GSE21422, GSE61304) datasets and TCGA BRCA mRNA data to screen for the common DEGs. As a result, 34 genes were identified and subsequently performed to assess the biological function of the DEGs, suggesting there were 18 genes mostly involved in cell cycle process. We further selected “TCGA-BRCA” cohort with abundant clinical characteristic information for validation the prognosis-related DEGs, and three crucial genes (TPX2, UBE2C and CCNE2) viewed as the common member genes based on the intersection analysis of the prognosis-related DEGs dataset and CCRGs.

Then, we systematically examined the clinical stage and survival analysis of the above three genes, and chose TPX2 as the crucial molecular to further exploration. As a microtubule-associated protein, TPX2 was essential for normal assembly of mitotic spindles and related to cell cycle and proliferation [12]. In the previous studies, TPX2 has been demonstrated as an important biomarker for predicting the evolution of some specific tumors, such as hepatocellular carcinoma and lung adenocarcinoma. Herein, we also found that TPX2 was overexpressed in BRCA and acted a detrimental prognostic factor. Furthermore, the expression of TPX2 in different stages was significantly different and suggested that TPX2 might be involved in BRCA progression to varying degrees at different stages.

Immunotherapy has been shown to prolong survival in patients with various solid tumors, and past evidence suggests that breast cancer is a low-immunogenic malignancy distinct from highly immunogenic tumors such as non-small cell lung cancer, malignant melanoma, etc. However, recent studies of TILs and tumor genome sequences have shown that breast cancer also has a potential immune response [37]. Therefore, searching and exploiting potential immunotherapy-relevant biomarkers is urgent. In this study, we found that the expression level of TPX2 was closely associated with B cell naïve, CD4 T cells, regulatory T cells, macrophages and mast cells. Interestingly, the transcription level of TPX2 was positively related with the abundance of CD4 T cells; yet, the methylation level of TPX2 was inversely correlated with that of CD4 T cells, suggesting TPX2 methylation might change the immunogenic role of TPX2 in BRCA. Besides, we discovered TPX2 had more or less relations with these representative immune checkpoints, which further proved that TPX2 was a potential immunogenic biomarker.

Previous reports have emphasized that TPX2 always assembled with Aurora Kinase A (AURKA) through its own phosphorylation to regulate the establishment of mitotic spindle, thereby affecting the process of cell cycle and tumorigenesis [38]. In addition, the prerequisite for AURKA to remain active was that TPX2 was required to stimulate its active site [39, 40]. Therefore, TPX2 could be viewed as an indispensable barometer for the regulation of important biological activities like chromosome assembly and spindle separation. Interestingly, AURKA has demonstrated that elevated PD-L1 expression and triggers PD-L1-mediated immune suppression in triple-negative breast cancer [41, 42]. So, we also chosen the most frequently studied cell MDA-MB-231 to verify the relationship of TPX2 and PD-L1, besides, the tissue chip also confirmed the positive correlation between them. However, the mechanism of TPX2 mediated immunosuppression or activation needs further experimental investigation.

In summary, our results identified that TPX2 as the most promising prognostic biomarker from the intersection analysis of TCGA and GEO database, and discovered that TPX2 was connected with some clinical features, such as T stage, N stage, age and histological type. In addition, we also investigated its relationship with immune components and further verified the positive correlation with PD-L1, which may conduct a new promising direction for immunotherapy in TNBC. All in all, we are trying to explore the mechanism of TPX2 on immune cells in breast cancer, which will be the direction and focus of our future efforts, aiming for exploring more powerful strategies targeting breast cancer patients.

## Supplementary Material

Supplementary Figures

Supplementary Tables
