# Supplementary material for: Integrative analysis based on the cell cycle-related genes identifies TPX2 as a novel prognostic biomarker associated with tumor immunity in breast cancer
Source: Aging (Albany NY). 2024 Apr 19;16(8):7188–216. doi: 10.18632/aging.205752 (PMC11087105; doi:10.18632/aging.205752)
Supplement: Supplementary Figures [file aging-16-205752-s001.pdf]

SUPPLEMENTARY FIGURES

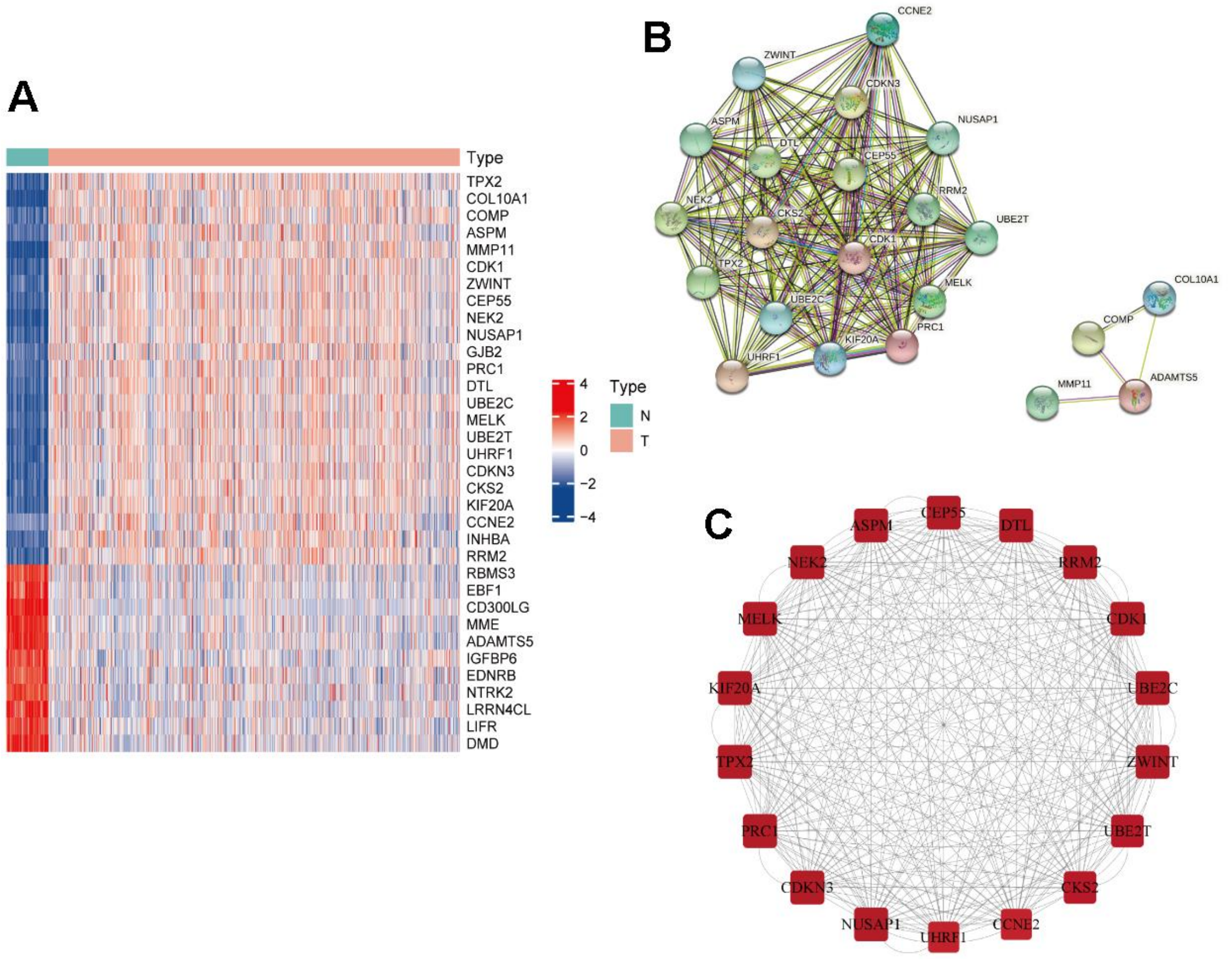

Supplementary Figure 1. PPI network construction and module analysis. (**A**) The heatmap illustrating the expression landscape of these DEGs in tumor and normal samples. (**B**) PPI network of these 34 DEGs. Nodes that were not associated with other nodes were hidden. (**C**) Extraction crucial module by MCODE plugin. Red dots represent interaction genes with a level of high degree.

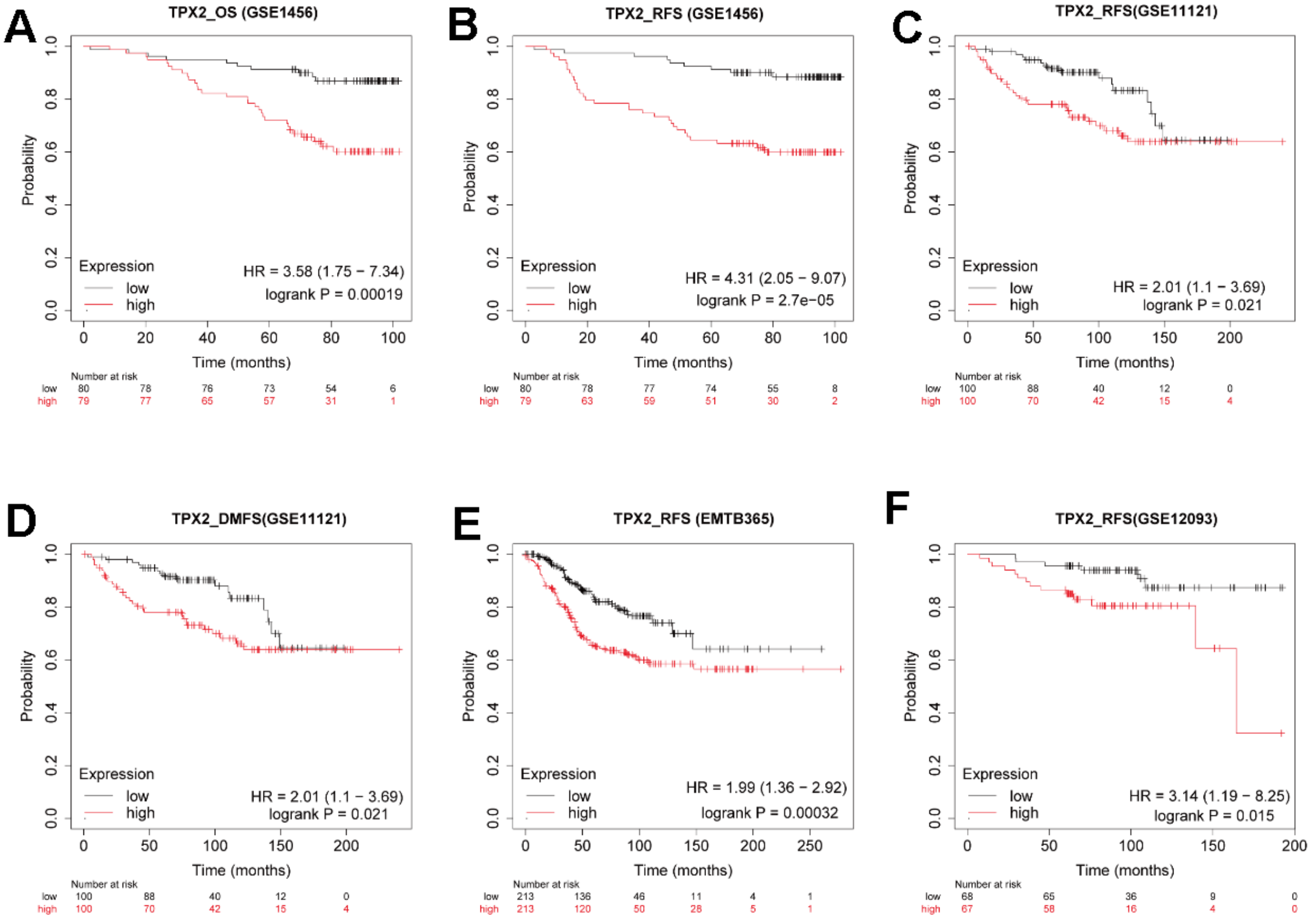

Supplementary Figure 2. The survival analysis of TPX2 with overall survival (OS), recurrence free survival (RFS), distant Metastasis Free Survival (DMFS) indicators respectively through various datasets (**A**–**F**). P <0.05 was considered significant.

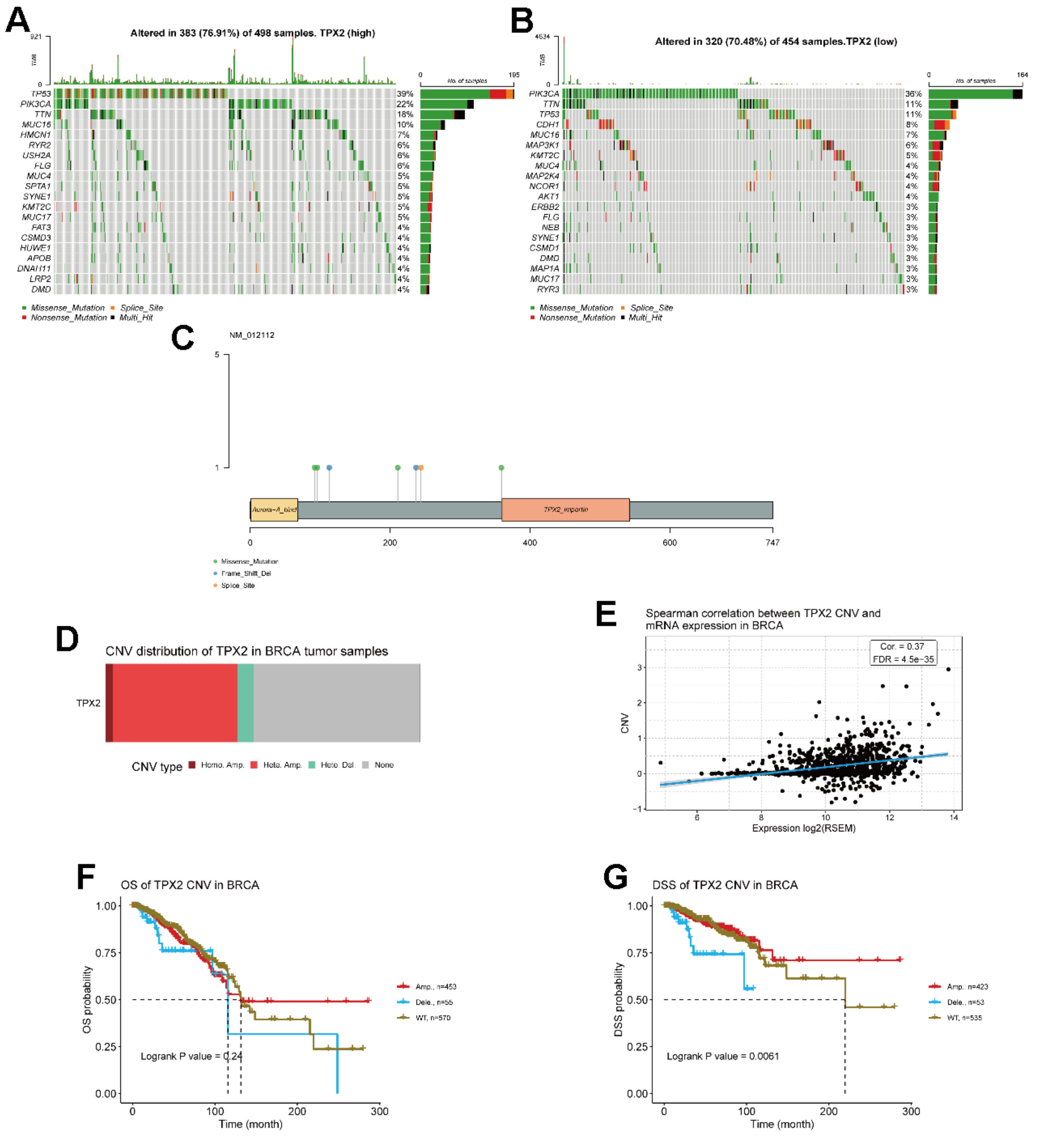

Supplementary Figure 3. The mutation landscape of TPX2 in TCGA-BRCA dataset. (**A**, **B**) The differential mutation genes in high TPX2 expression samples (n=498) and low TPX2 expression samples (n=454). (**C**) The mutation sites of TPX2 gene, different dots represent various mutation form. Green represents missense mutation, blue represents frame shift del, orange represents splice site. (**D**) The CNV distribution of TPX2 in BRCA tumor samples. (**E**) The correlation between TPX2 CNV and mRNA expression in BRCA. (**F**, **G**) The survival analysis of TPX2 CNV with overall survival (OS) and disease specific survival (DSS) indicators via TCGA-BRCA dataset. P<0.05 was considered significant.

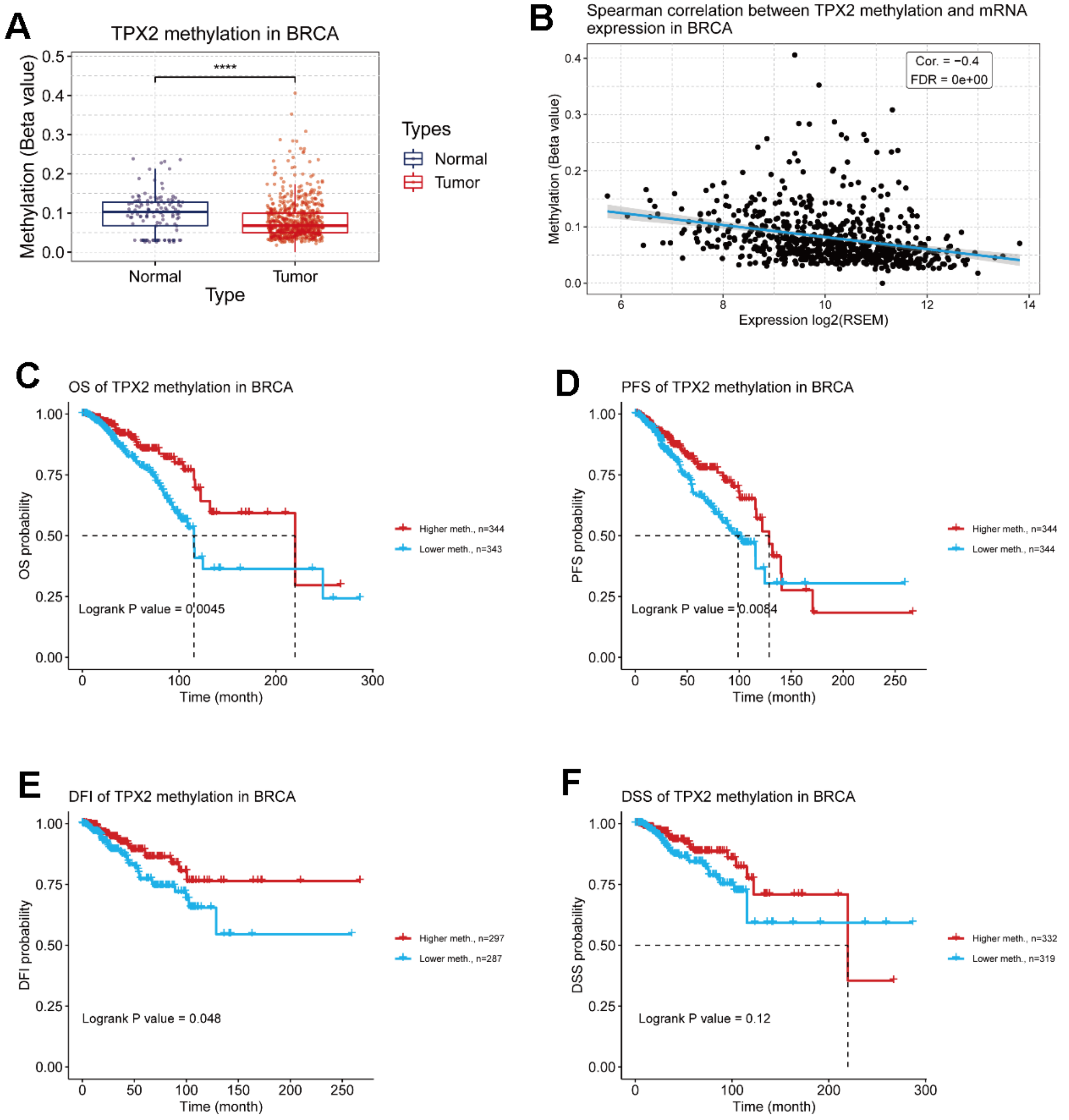

Supplementary Figure 4. The methylation level of TPX2 in TCGA-BRCA dataset and its relationship with prognosis. (**A**) The methylation of TPX2 in BRCA dataset. (**B**) The correlation between TPX2 methylation and mRNA expression. (**C**–**F**) The survival analysis of TPX2 methylation with overall survival (OS), progression free survival (PFS), disease free interval (DFI), disease specific survival (DSS) indicators, respectively. P<0.05 was considered significant.

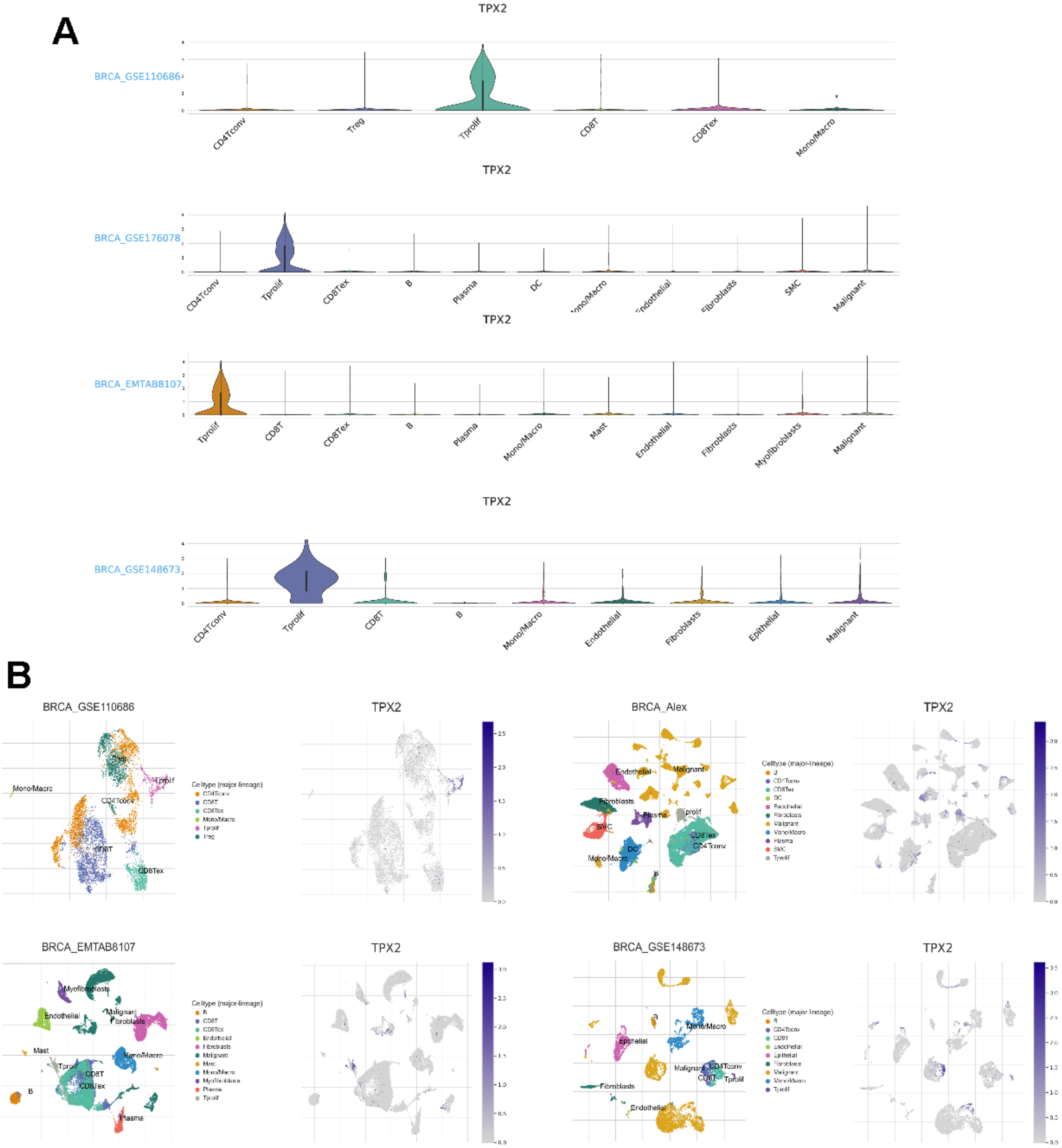

Supplementary Figure 5. The distribution of TPX2 expression in different cell types from the four GEO datasets. (**A**) The Violin plot illustrating the association between TPX2 expression and various cell types in each GEO dataset. (**B**) The t-SNE plot showing the cell types identified in BRCA with different colors, and performing the distribution of TPX2 in these cells.

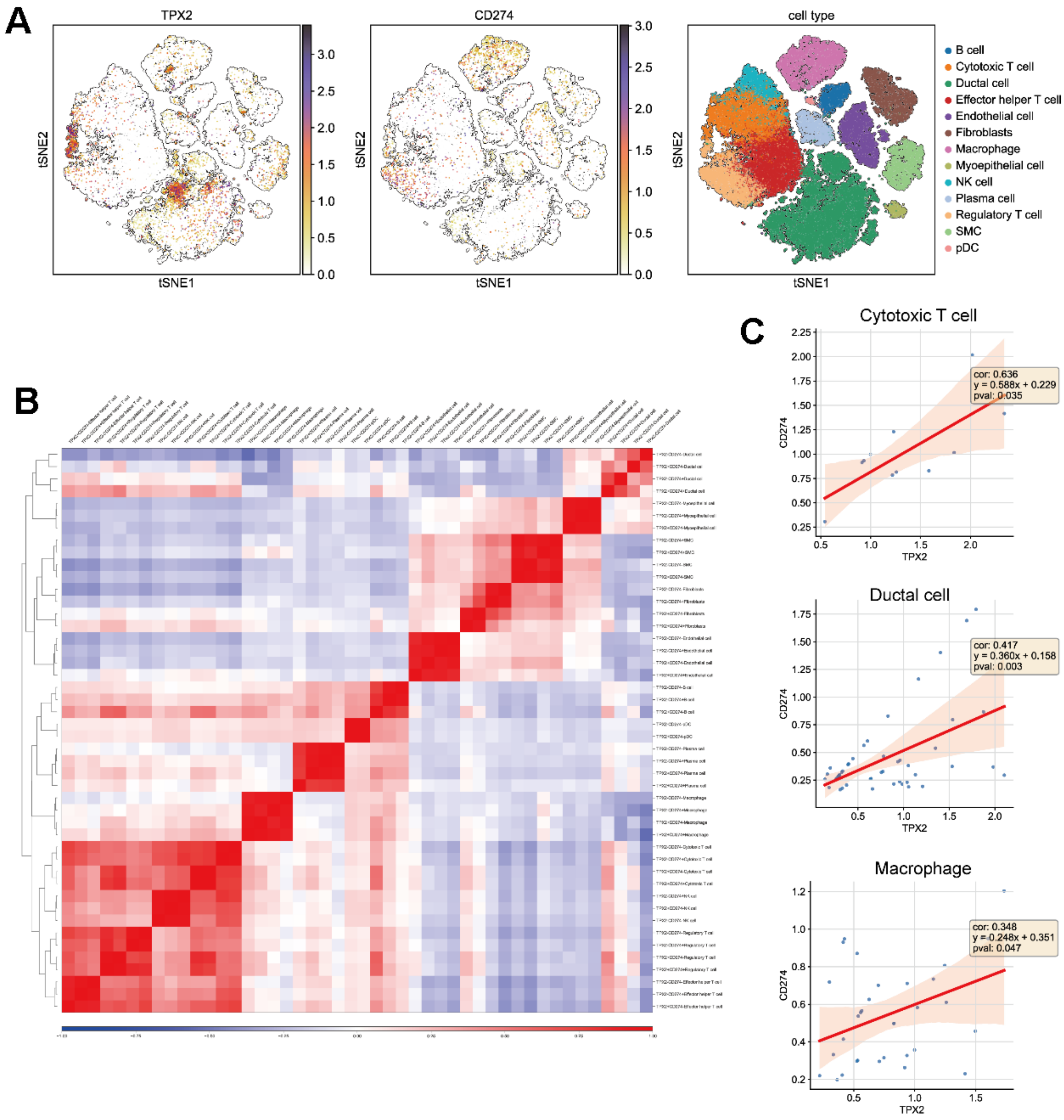

Supplementary Figure 6. (**A**) The t-SNE plot showing the cell types identified in BRCA with different colors, and performing the distribution of TPX2 and CD274 in these cells through GSE176078 single cell dataset. (**B**) The heatmap illustrating the association between TPX2 and CD274 in different cells. (**C**) The correlation analysis between TPX2 and CD274 in cytotoxic T cell, ductal cell and macrophage cell.

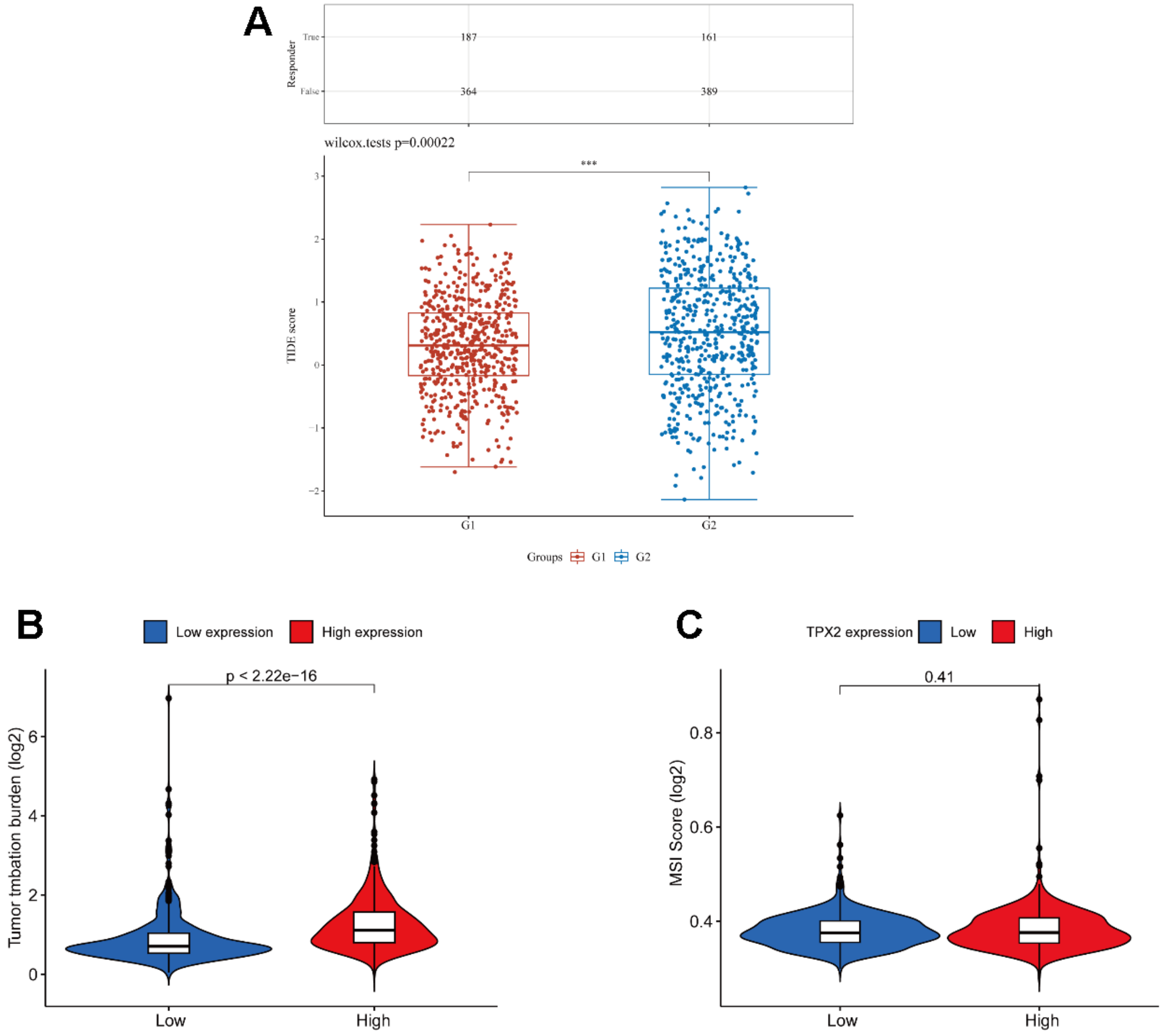

Supplementary Figure 7. (**A**) The relationship between TPX2 expression and immune response and TIDE score. G1: TPX2-high, G2: TPX2-low. (**B**, **C**) The correlation analysis between TPX2 expression and TMB and MSI.
