# Supplementary material for: Integrative analysis based on the cell cycle-related genes identifies TPX2 as a novel prognostic biomarker associated with tumor immunity in breast cancer
Source: Aging (Albany NY). 2024 Apr 19;16(8):7188–216. doi: 10.18632/aging.205752 (PMC11087105; doi:10.18632/aging.205752)
Supplement: Supplementary Tables [file aging-16-205752-s002.pdf]

SUPPLEMENTARY TABLES

Supplementary Table 1. Details of the highest node degree of DEGs.

Name	logFC	Function
ASPM	3.5316443528509	mitotic spindle regulation
MELK	3.63564169071688	cell cycle regulation; self-renewal of stem cells; apoptosis and splicing regulation; protein kinase activity
TPX2	3.56995205916141	assembly of mitotic spindles; cell cycle regulation; microtubule nucleation; protein kinase activator activity
CDKN3	2.90721214654034	cell cycle regulation; protein tyrosine phosphatase activity
UBE2C	4.18668130704087	ubiquitin-protein transferase activity
RRM2	3.40878569224278	oxidoreductase activity; ribonucleoside-diphosphate reductase activity; DNA synthesis
DTL	3.04451639061984	ubiquitin-protein transferase activity; protein-macromolecule adaptor activity; cell cycle control; DNA damage response; DNA synthesis
NEK2	4.24646669738718	protein kinase activity; centrosome duplication and separation; microtubule stabilization; kinetochore attachment; spindle assembly checkpoint
ZWINT	2.58065857349798	mitotic spindle checkpoint; kinetochore attachment
CEP55	3.42973503811904	cytokinetic abscission; cytokinesis process regulation
KIF20A	3.37521864678303	microtubule motor activity
PRC1	2.64789579661942	cytokinesis process regulation
NUSAP1	2.64044477064212	microtubule stabilization
CDK1	3.15252348887215	centrosome cycle modulation; protein kinase activity; virus receptor activity; cell cycle regulation

Supplementary Table 2. TPX2 expression and survival data of breast cancer patients using the PrognoScan database.

Dataset	Endpoint	Probe ID	Number	COX P-value	HR
GSE7378	DFS	210052_s_at	54	0.002655	2.83 [1.44 - 5.58]
GSE9195	DMFS	210052_s_at	77	0.008593	2.56 [1.27 - 5.17]
GSE1456	RFS	210052_s_at	159	0.000043	2.23 [1.52 - 3.27]
GSE1456	OS	210052_s_at	159	0.000682	1.95 [1.33 - 2.87]
GSE3143	OS	210052_s_at	158	0.007631	1.92 [1.19 - 3.09]
GSE3494	DSS	210052_s_at	236	0.001959	1.74 [1.23 - 2.47]
GSE4922	DSS	210052_s_at	249	0.001589	1.55 [1.18 - 2.04]
GSE2990	RFS	210052_s_at	62	0.022996	1.87 [1.09 - 3.20]
GSE6532	DMFS	210052_s_at	87	0.009029	1.64 [1.13 - 2.38]
GSE4922	DFS	210052_s_at	249	0.001589	1.55 [1.18 - 2.04]

Supplementary Table 3. Clinical information of BRCA patients and association analysis between TPX2 expression and these clinical features.

Variables	Number	Percentage (%)	P-value
**Age**			0.6604
<60	116	80.0	
≥60	29	20.0	
**T stage**			**0.0112**
T1-T2	92	63.9	
T3-T4	52	36.1	
**N stage**			0.3302
N0-N1	120	82.8	
N2-N3	25	17.2	
**Stage**			**0.0208**
I+ II	91	63.2	
III	53	36.8	
**Grade**			**<0.0001**
I+ II	101	69.7	
III	44	30.3	
**Subtype**			0.0542
Non-TNBC	104	71.7	
TNBC	41	28.3	

Statistically significant ($P < 0.05$) values are in bold. TNBC, triple-negative breast cancer.

Supplementary Table 4. Correlation analysis between TPX2 and related genes and markers of immune cells in TIMER.

Description	Gene markers	BRCA			
		None		Purity	
		Cor	P	Cor	P
CD8+ T cell	CD8A	0.064	*	0.206	***
	CD8B	0.061	*	0.192	***
B cell	CD19	0.048	ns	0.163	***
	CD79A	0.022	ns	0.15	***
Monocyte	CD86	0.155	***	0.254	***
	CD115 (CSF1R)	-0.104	***	-0.021	ns
TAM	CCL2	0.111	***	0.207	***
	CD68	0.144	***	0.226	***
	IL10	0.205	***	0.293	***
M1 Macrophage	INOS (NOS2)	0.067	*	0.08	*
	IRF5	0.12	***	0.178	***
	COX2 (PTGS2)	0.005	ns	0.089	**
M2 Macrophage	CD163	0.211	***	0.297	***
	VSIG4	-0.012	ns	0.057	ns
	MS4A4A	0.102	***	0.21	***
Neutrophils	CD66b (CEACAM8)	0.039	ns	0.032	ns
	CCR7	0.053	ns	0.191	***
	CD11b (ITGAM)	0.011	ns	0.075	*
Th1	T-bet (TBX21)	0.075	*	0.219	***
	STAT4	0.039	ns	0.178	***
	STAT1	0.365	***	0.418	***
	IFN-γ (IFNG)	0.214	***	0.324	***
	TNF-α (TNF)	0.234	***	0.276	***
Th2	GATA3	-0.253	***	-0.33	***
	STAT6	-0.24	***	-0.223	***
	STAT5A	-0.213	***	-0.159	***
	IL-13	0.09	**	0.125	***
Treg	FOXP3	0.261	***	0.376	***
	TGFβ (TGFB1)	-0.26	***	-0.199	***
	CCR8	0.339	***	0.422	***
	STAT5B	-0.214	***	-0.187	***
T cell exhaustion	PD-1 (PDCD1)	0.108	***	0.24	***
	CTLA4	0.259	***	0.388	***
	LAG3	0.299	***	0.378	***
	TIM-3 (HAVCR2)	0.121	***	0.21	***
	GZMB	0.247	***	0.376	***

TAM, tumor-associated macrophage; Th, T helper cell; Treg, regulatory T cell; Cor, R value of Spearman's correlation; NS, correlation without adjustment; Purity, correlation adjusted by purity.

*P < 0.05.

**P < 0.01.

***P < 0.001.

Supplementary Table 5. Detailed information of the interaction between TPX2 and PD-L1.

Receptor (A)	Ligand (B)	Hydrogen bond interaction (2.5Å)[a]	Electrostatic interaction (4Å)
**TRX2 (6BJC)**	PDL1 (3BIK)	A:Gln11-B:Ala247<br>A:Val353-B:Val353<br>A:Gly100-B:Asn258<br>A:Phe404-B:Pro261<br>A:Trp407-B:Val260<br>A:Hie406-B:Val260<br>A:Lys189-B:Thr130	A:Glu411-B:Lys163

Supplementary Table 6. The interacted and correlated genes of TPX2.

Interacted genes		Correlated genes			
KIF11	BRIP1	KIF4A	CENP1	ORC1	CASC5
MAPRE3	BARD1	BUB1	MELK	CDC25A	HMMR
CENPJ	AURKA	PLK1	CDCA8	LMNB1	GMPS
TUBB2A	AURKB	KIF20A	CCNB1	SKA3	RAD51
STMN4	FZR1	CKAP2L	EXO1	RACGAP1	WDHD1
TUBB3	MYCN	DLGAP5	CENPE	UBE2C	CDKN3
DCX	INCENP	HJURP	KIF18B	CENPN	ANLN
TUBB	TACC3	NCAPH	CENPF	OIP5	PRC1
TUBA1B	CCNB1	ASPMEN	GINS1	NEIL3	CHEK1
KIF1A	CDC27	CENPA	AURKB	ESPL1	CDC25C
TUBA1A	ANAPC15	KIF23	GTSE1	SPC25	SUV39H2
TUBB2B	UBE2C	BUB1B	GSG2	KNSTRN	MTFR2
KIF2C	ANAPC2	NCAPG	CCNA2	IQGAP3	ARHGAP11A
KIF5A	CDC16	KIF15	CENPO	E2F1	MAD2L1
KIF5B	ANAPC13	KIF2C	NDC80	INCENP	FAM72A
KIF18A	FBXO5	SGOL1	KIF14	TROAP	MCM6
KIF2A	CDC23	KIF11	SKA1	MCM10	UBE2T
KIF7	ANAPC7	KIFC1	NEK2	SGOL2	PLK4
KIF5C	ANAPC5	CEP55	POLQ	GPSM2	SKP2
DYNC1H1	ANAPC1	ERCC6L	DEPDC1B	CDCA3	RAD54L
ACACA	ANAPC4	FAM83D	TRIP13	SMC4	PTTG1
BRCA1	ANAPC10	CCNB2	NUSAP1	FAM72B	GAS2L3
HMMR	CDC26	MKI67	PARPBP	MYBL2	CKS1B
ZNF598		KIF18A	STIL	ECT2	TUBB
RBBP8		TTK	FOXM1	MCM2	CDC45
